# Genome-wide mapping reveals conserved and diverged R-loop activities in the unusual genetic landscape of the African trypanosome genome

**DOI:** 10.1093/nar/gky928

**Published:** 2018-10-10

**Authors:** Emma Briggs, Graham Hamilton, Kathryn Crouch, Craig Lapsley, Richard McCulloch

**Affiliations:** 1 The Wellcome Centre for Molecular Parasitology, University of Glasgow, College of Medical, Veterinary and Life Sciences, Institute of Infection, Immunity and Inflammation, Sir Graeme Davies Building, 120 University Place, Glasgow G12 8TA, UK; 2 Glasgow Polyomics, University of Glasgow, Wolfson Wohl Cancer Research Centre, Garscube Estate, Switchback Rd, Bearsden, G61 1QH, UK

## Abstract

R-loops are stable RNA–DNA hybrids that have been implicated in transcription initiation and termination, as well as in telomere maintenance, chromatin formation, and genome replication and instability. RNA Polymerase (Pol) II transcription in the protozoan parasite *Trypanosoma brucei* is highly unusual: virtually all genes are co-transcribed from multigene transcription units, with mRNAs generated by linked trans-splicing and polyadenylation, and transcription initiation sites display no conserved promoter motifs. Here, we describe the genome-wide distribution of R-loops in wild type mammal-infective *T. brucei* and in mutants lacking RNase H1, revealing both conserved and diverged functions. Conserved localization was found at centromeres, rRNA genes and retrotransposon-associated genes. RNA Pol II transcription initiation sites also displayed R-loops, suggesting a broadly conserved role despite the lack of promoter conservation or transcription initiation regulation. However, the most abundant sites of R-loop enrichment were within the regions between coding sequences of the multigene transcription units, where the hybrids coincide with sites of polyadenylation and nucleosome-depletion. Thus, instead of functioning in transcription termination the most widespread localization of R-loops in *T. brucei* suggests a novel correlation with pre-mRNA processing. Finally, we find little evidence for correlation between R-loop localization and mapped sites of DNA replication initiation.

## INTRODUCTION

RNA–DNA hybrids display enhanced stability compared with double-stranded DNA or RNA due to the unusual conformation they adopt (1,2). Small RNA–DNA hybrids form during priming of DNA replication and within RNA polymerase (Pol) during transcription, whereas larger RNA–DNA hybrids, termed R-loops, can form when RNA exiting the RNA Pol can sometimes access the DNA before the duplex reanneals. These R-loops are exacerbated when elements of RNA biogenesis are impaired (3–7) and are increasingly recognized as providing widespread roles (8–10), which are not all co-transcriptional. R-loops may also form in *trans*, when RNA moves from the site of its genesis to another, homologous location. One example of *trans* R-loop formation is seen during the prokaryotic CRISPR-cas defence system (11). In addition, eukaryotic and bacterial homologous recombination proteins (which normally direct DNA repair) are capable of generating RNA–DNA hybrids (12–15).

R-loops can be detrimental to genome function, leading to instability and mutation (16–18), for instance by blocking replication or because of increased lability of the exposed single-stranded DNA. In addition, sites of DNA replication and transcription collision have been shown to accumulate R-loops (19,20). To counteract these detrimental effects, activities have been described in eukaryotic cells to limit R-loop formation during mRNA biogenesis (8,21). In addition, all cells encode activities to resolve R-loops once they form. Beyond a number of helicases that can unwind R-loops (8,21), RNase H enzymes play a key role in all cells in degrading the RNA within the hybrid. Most prokaryotes and eukaryotes encode two RNase H enzymes (22): one, eukaryotic RNase H1, is monomeric and appears conserved with bacterial RNase HI, while RNase H2 in eukaryotes is trimeric and therefore differs structurally from monomeric bacterial RNase HII. Distinct roles might be predicted by the presence of two RNase H enzymes throughout life, but separate R-loop-associated functions in the eukaryotic nucleus have been hard to identify, though RNase H2 is additionally able to excise ribonucleotides mis-incorporated into DNA (23), while RNase H1 acts on organelle genome R-loops in yeast, mammals and plants (24–26). Loss of either RNase H is lethal in mammals (25,27), but yeast mutants lacking both enzymes are viable and display increased nuclear R-loop abundance (6,24,28). Clashes between transcription and replication can be resolved in bacteria by RNase HII acting on the resulting R-loops (19), while the R-loops that form in the same circumstances in eukaryotes have been shown to lead to DNA damage signalling (20) and the recruitment of repair factors (29–31).

Growing evidence suggests that R-loops are not always detrimental, but instead are increasingly associated with functional roles. Translocations in the mammalian immunoglobulin locus can cause lymphoma, but at least some of these cancers may be an unfortunate by-product of the deliberate generation of R-loops to mediate immunoglobulin class switch recombination during B-cell maturation (32,33). R-loops also have a key role in initiating replication of bacterial plasmids (34), mitochondrial DNA (35) and phage genomes (36). Indeed, it has been suggested R-loops contribute to DNA replication initiation at cryptic bacterial origins (37) and to at least some eukaryotic nuclear DNA replication initiation (38,39). Beyond recombination and replication, gene expression control can be enacted by R-loops (8,10). In human cells, R-loops at CpG island promoters protect against epigenetic silencing (40–42), while in *Arabidopsis* the hybrids may promote silencing of some genes (43). Similarly, in yeast and mammalian cells R-loop association with chromatin modification and non-coding RNA mediates termination of transcription (44–47). Indeed, R-loop and chromatin modification might be a widespread association (48–50), since the hybrids can be found throughout some protein-coding genes, rather than being limited to promoters and terminators (51,52). Finally, TERRA RNAs generated by the transcription of the telomere repeats at the ends of eukaryotic chromosome can form R-loops (53), which may provide a means to maintain telomeres in the absence of telomerase, including through recombination (54,55).

The above, widespread localization of R-loops has only to date been explored in organisms that follow conventional rules for eukaryotic protein-coding gene expression, where each protein is normally found as a single transcription unit with its own promoter and terminator. Gene expression in kinetoplastids, a grouping of protozoans that includes several major human and animal parasites, does not conform to these rules (56). One such kinetoplastid is *Trypanosoma brucei*, and co-ordination of gene expression here largely reflects the broader range of organisms in this lineage. Virtually every protein-coding gene (∼8000) in *T. brucei* is transcribed from a small number (∼200) of multigene transcription units, with little evidence for functional grouping within the units (57). Only two genes have been described to have introns (58) and mature mRNAs are generated from multigene RNA transcripts by coupled *trans*-splicing and polyadenylation (59). Remarkably, some protein-coding genes in *T. brucei* are expressed from multigene units transcribed by RNA Pol I, where the promoters share some homology with those at rRNA gene clusters (60). The vast majority of protein coding genes are more conventionally transcribed by RNA Pol II, but from promoters that are still not fully understood. Sites of transcription initiation have been mapped to so-called strand switch regions (SSRs) that separate adjacent RNA Pol II transcription units (61,62), but conserved sequence motifs characteristic of eukaryotic RNA Pol II promoters have escaped detection. Instead, it appears increasingly likely that SSRs are more similar to dispersed and unregulated eukaryotic RNA Pol II promoters (63), consistent with a lack of control over transcription initiation and devolution of gene expression controls to post-transcriptional reactions, such as mRNA turnover (64). Transcription termination at the ends of multigene transcription units in *T. brucei* is even more poorly understood, though variant and modified histones have been localized to terminator SSRs, as has a modified base, called J (65,66). In fact, SSRs are not merely sites of transcription initiation, but are also the locations where DNA replication initiates, termed origins (67,68). Functional interaction between the replication and transcription machineries is suggested by RNA changes around the SSRs after RNAi against ORC1/CDC6, a subunit of the origin recognition complex, which binds all SSRs but directs DNA replication initiation (69) at only a subset of ∼45 SSRs (67,68). What features distinguish origin-active SSRs from non-origin SSRs is unclear, but the wide separation of origins and their co-localization with some SSRs may limit deleterious collisions between the DNA and RNA Pol machineries in the context of multigenic, pervasive transcription (67,70).

The highly divergent genetic landscape found in the *T. brucei* genome provides an excellent platform to ask what features of R-loop localization and function are conserved across eukaryotes and to potentially reveal kinetoplastid or *T. brucei*-specific roles. Localization of R-loops has been greatly aided by the generation of the monoclonal antibody S9.6, which binds RNA–DNA hybrids (71) and, to a lesser extent, double-stranded RNA (72). Here, we used S9.6 for RNA–DNA hybrid immunoprecipitation (IP) to evaluate R-loop distribution genome-wide in both wildtype bloodstream form (BSF, mammal-infective) *T. brucei* cells and in null mutants lacking RNase H1. We show that R-loops are very abundant across the *T. brucei* genome, with the most pronounced genomic localization seen throughout the RNA Pol II multigene transcription units, where R-loops coincide most clearly with polyadenylation sites and regions of nucleosome depletion. Thus, in these locations we reveal a novel association of R-loops, which is not related to transcription termination, but potentially to pre-mRNA processing and/or ensuring a chromatin landscape needed for the continued movement of RNA Pol. In addition, despite the divergence of *T. brucei* RNA Pol II promoters, we show significant enrichment of R-loops at sites of transcription initiation, indicating promoter-proximal RNA–DNA hybrids are not merely associated with the regulation of transcription but may be necessary for the mechanics of initiation. Beyond these novel R-loop associations, we reveal widespread conserved localization to retrotransposon-associated sequences, rRNA and centromeres, but can find no evidence that R-loops preferentially form at DNA replication origins in *T. brucei*.

## MATERIALS AND METHODS

### *T. brucei* cell lines

All cell lines used were BSF *T. b. brucei*, strain Lister 427, and were maintained in HMI-9 medium supplemented with 10% (v/v) FBS (Sigma-Aldrich, Missouri, USA) and 1% (v/v) penicillin-streptomycin solution (Gibco) at 37°C and 5% CO2. Heterozygous (–/+) and homozygous (–/–) *Tbrh1* knockout cell lines were generated using two constructs containing cassettes of either blasticidin or neomycin resistance genes between α-β tubulin and actin intergenic regions, flanked by sequences homologous to the 5′ and 3′ UTRs of *TbRH1*, essentially as described in (Devlin et al 2016). Homologous flanking regions were PCR-amplified using the following primers: 5′ UTR CGACG*GGATCC*TTGCCTTACCCGTGTTTT and CGACG*TCTAGA*CCTTTTCTTTCCCATGGAC, 3′ UTR CGACG*CCCGGG*AGGTGTGTATGGGAATGA and CGACG*CTCGAG*GCACCACCCAGTATAGAAA.

### DRIP analysis

DRIP was performed using a ChIP-IT Enzymatic Express kit (Active Motif). Briefly, ∼2 × 10^8^ cells were grown to log phase before fixing in 1% formaldehyde for 5 min whilst shaking at room temperature, before 1 ml of 10× glycine buffer was added directly to the cells to stop fixation. Cells were then pelleted, re-suspended in Glycine Stop-Fix Solution and shaken at room temperature for 5 min. Cells were next lysed, according to the manufacturer's protocol, allowing chromatin to be extracted and digested for 5 min with Enzymatic Shearing Cocktail at 37°C to produce ∼200 bp fragments. IP was performed overnight at 4°C with 4.5 ng of S9.6 antibody (Kerafast). For DRIP-qPCR analysis each replicate chromatin sample was divided in two, allowing parallel IP reactions to be performed, one of which was subjected to *Escherichia coli* RNase HI treatment, essentially as described in (24).

Library preparation was performed using a TruSeq ChIP Library Preparation Kit (Illumina) and fragments of 300 bp, including adaptors, were selected with Agencourt AMPure XP (Beckman Coulter). Sequencing was performed with an Illumina NextSeq 500 platform. Reads were trimmed using TrimGalore (https://github.com/FelixKrueger/TrimGalore) under default settings before alignment to a ‘hybrid’ reference genome consisting of the TRUE-927 v5.1 core chromosome assembly, plus sequences of 14 Lister 427 VSG ES and 5 mVSG ES (73) using Bowtie2 (74) in ‘very-sensitive’ mode. Reads with a MapQ value <1 were removed using SAMtools (75), leaving at least 30 million aligned reads per sample. The fold-change between input and DRIP read depth was determined for each sample over non-overlapping 50 bp windows using the DeepTools bamCompare tool: library size was normalized via the SES method, foldchange was expressed as a ratio, and data visualized as tracks with IGV (76). Regions with a fold-change ≥1.2 were considered enriched and adjacent enriched windows were combined to give the coordinates of final DRIP enriched regions. No gaps were allowed between regions.

Classification was accomplished by assessing DRIP enriched region coordinate overlap with different genomic regions: VSG subtelomeric arrays, RHS, centromeres, Pol I, Pol II and Pol III transcripts, SSRs, mVSG and VSG ES. Each DRIP enriched region was assigned to the genomic region for which it showed the greatest overlap. Enriched regions which displayed no overlap with any feature were assumed to locate within the Pol II PTUs. Enriched regions assigned to the Pol II PTUs were further classified as associated with the CDS or UTR sequences, or else assigned as intergenic. Motif analysis of Pol II PTU-associated enriched regions was done using MEME version 4.12.0 under default settings (77).

Normalized ratio bigwig DRIP-seq files were used to generate metaplots and heatmaps using deepTools (78). DRIP versus PAS metaplots were generated with the makemetaplot.pl script from HOMER using PAS coordinates (61,79) and enriched region coordinates. H3.V, H4.V, H2A.V, H2B.Z, H4k10ac and BDF3 ChIP-seq data was sourced from (62), H3 ChIP-seq from (63) and mRNA half-life from (64). All data were processed as per publication methods. R-loop predications were made using the QmRLFS-finder algorithm (80) that is based upon the findings by (81), where an R-loop forming sequencing (RLFS) was defined as containing three parts: an initiation zone containing G-clustering, a G-rich elongation zone where the R-loop can extend, and a linking sequence between 0 and 50 bp of any composition. In this analysis G-clustering was defined as three clusters containing ≥3 G residues, or two clusters of ≥4 G residues. In both cases clusters were separated by 1–10 bp. Elongation zones were defined as comprising ≥40% G residues and 100–2000 bp in length.

### Quantitative PCR

In the case of DRIP-qPCR, DRIP samples were prepared as for DRIP-seq above. For RT-qPCR, RNA extracts were made with 1 × 10^7^ parasites using the RNeasy Mini Kit (Qiagen^®^) and cDNA was generated with the SuperScript™ First-Strand Synthesis System (Invitrogen, Life Technologies) using random hexamers. In both cases 21 μl qPCR reactions were set up with 10 μl SYBR™ Select master mix (Applied Biosciences™), 350 nM each primer and 1 μl diluted DRIP/cDNA sample. qPCR was run with the 7500 Real Time PCR system (Applied Biosystems®) as follows: 10 min at 95°C, 40 cycles of 95°C for 15 s and 60°C for 1 min. Relative mRNA fold-change between WT and *Tbrh1–/–* samples was calculated with the 2^–ΔΔCt^ method (82). For DRIP-qPCR, Ct values were first correct for dilutions (minus log2 of dilution factor) and input percentage was calculated as 100 × 2 ^(input-IP)^.

### RNA-seq analysis

For RNA-seq, total RNA was extracted using the RNeasy Mini Kit (Qiagen), and poly(A) selection and library preparation was performed using the TruSeq Stranded Total RNA kit (Illumina). 75 bp paired-end reads were generated using an Illumina NextSeq 500 platform. After trimming with Trim Galore, reads were aligned to a ‘hybrid’ reference genome (core and subtelomeres from TREU927, VSG ES from Lister 427; gift, S. Hutchinson). Alignment was carried out using Hisat2 (–no-spliced-alignment) (83) and reads with a MapQ value <1 were removed using SAMtools as previously described (73). Uniquely aligned reads on the coding strand of each exon were counted using HTSeq-count (-s reverse, union mode) [https://htseq.readthedocs.io/en/release_0.10.0/count.html]. Counts were normalized between samples using the regularized log expression method as implemented in DESeq2 (84) and normalized counts from *Tbrh1*–/– and WT samples were plotted against each other.

### GC and AT skew analysis

GC and AT skew were calculated as (G-C)/(G+C) and (A-T)/(A+T), respectively. Skew was calculated either in 11 bp windows (analysis of ATG translational start sites only) or in 100 bp windows across the *T. brucei* hybrid genome. The results of whole genome analysis were converted into bigwig format and skew was plotted over regions of interest with deepTools analysis software.

## RESULTS

### R-loops are highly abundant in the *T. brucei* genome

RNA–DNA hybrid immunoprecipitation (DRIP), either on cross-linked or ‘naked’ DNA, has been used in a number of organisms, with some experimental variations based on treatment of the nucleic acid components, the mode of genome fragmentation and whether the recovered nucleic acid was analysed by sequencing, microarray hybridization or qPCR (85). Here, we used S9.6 for DRIP after formaldehyde fixation, an approach that has been applied to R-loop mapping in *Saccharomyces cerevisiae* (12,28,86,87), *Schizosaccharomyces pombe* (88) and *Arabidopsis thaliana* (89). To provide a genome-wide picture of R-loop distribution, DNA was isolated from the DRIP material and characterized by Illumina sequencing (DRIP-seq), mapping the reads to the assembled *T. brucei* genome (90). To compare R-loop distribution in cells with and without some of the factors that target RNA–DNA hybrids, DRIP-seq was performed, in duplicate, in both wild type (WT) BSF *T. brucei* cells and in null mutants lacking RNase H1 (TbRH1, encoded by gene Tb427.07.4930), allowing comparison of the mapping profiles. TbRH1 mutants were generated by two rounds of allele replacement, with the resulting *Tbrh1*–/– null cells found to be viable and to grow in culture at the same rate as WT BSF cells (Briggs *et al.*, BioRxiv https://doi.org/10.1101/361451).

**Figure 1.**
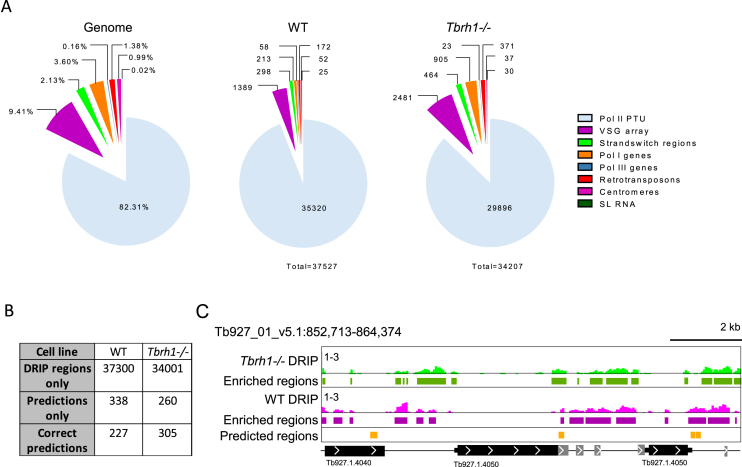
Distribution of R-loops in the genome of bloodstream form *T. brucei*. (**A**) Analysis of R-loop locations in WT and *Tbrh1–/–* cells showing DRIP enriched region data sets (middle and right charts) relative to the sequence composition of the 11 *T. brucei* 11 megabase chromsomes (left chart); genomic elements are colour-coded according to right-hand key. (**B**) Comparison of the number of WT and *Tbrh1–/–* DRIP enriched regions and predicted R-loop forming sequences. (**C**) Screenshot of an RNA Pol II transcribed region of chromosome 1. WT and *Tbrh1–/–* DRIP data is shown in pink and green, respectively (1–3 on y-axes denotes level of enrichment in DRIP relative to input), with identified enriched regions from each data set shown below, and predicted R-loop forming regions in orange. CDS are shown as thick black lines, UTRs as thin black lines and arrows show direction of transcription; size is indicated. Genes annotated as ‘hypothetical, unlikely’ are shown in grey.

DRIP-seq mapping to the *T. brucei* genome revealed very widespread coverage (Figure 1): based on >1.2-fold enrichment of reads in the DRIP sample relative to input, ∼35 000 enriched regions were predicted in both WT and *Tbrh1*–/– cells. To understand if the enrichment was localized to specific sequences, we divided the genome into regions transcribed by the three RNA Pols, largely non-transcribed VSG arrays and SSRs, and centromeric and retrotransposon and associated sequences (Figure 1A). DRIP-seq enriched regions were found in all these locations, but with notably highest abundance in the RNA Pol II polycistronic transcription units (PTUs; Figure 1A). Though loss of TbRH1 did not increase the number of enriched regions, greater abundance of putative R-loops were found in non-PTU regions (Figure 1A) and a greater amount of the genome was enriched (Supplementary Figure S1), consistent with spreading of R-loops following loss of the RNase H1 enzyme.

### R-loops are enriched at repeated sequences, including centromeres, RNA Pol I arrays and retrotransposon-associated genes

**Figure 2.**
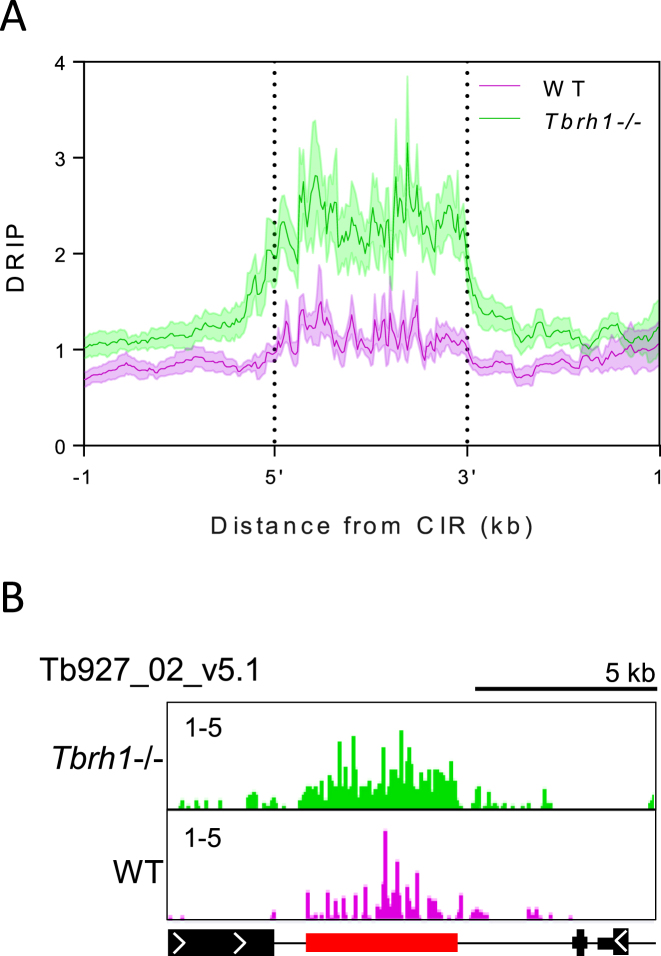
R-loops are enriched at *T. brucei* centromeres. (**A**) Metaplot of DRIP signal in WT (pink) and *Tbrh1–/–* cell (green) data sets centred on the annotated centromeric interspersed repeats (CIR) ±1 kb. (**B**) Representative screenshot of a portion of chromosome 2 (Tb927_02_v5.1) containing the centromere region; CDS and DRIP-seq enrichment annotations are shown as in Figure 1, CIR are shown in red.

**Figure 3.**
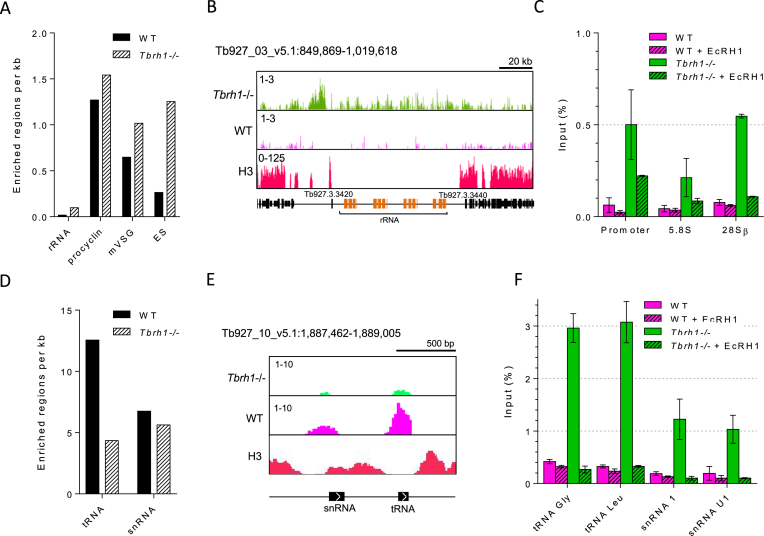
DRIP-seq signal at RNA Pol I and Pol III transcribed sites in *T. brucei*. (**A**) Graph showing the number of enriched regions identified per kb at Pol I transcribed loci for WT and *Tbrh1–/–* data sets. (**B**) A representative screenshot of a rRNA locus is shown, along with histone H3 ChIP-seq data (Wedel *et al.* 2017) (right; annotation as in Figure 1C). (**C**) Graph showing DRIP-qPCR targetting three regions of the rRNA locus; RNA Pol I promoter, 5.8S coding region and 28.S coding region. Pecentage of input sequence also detected in IP samples is shown for WT (pink) and *Tbrh1–/–* (green) cells. In each case EcRHI treated controls are shown as lined bars. Error bars shown SEM of two indepentent replicates. (**D**) Graph showing the number of enriched regions identified at Pol III transcribed loci. (**E**) A representative screenshot of a locus containing snRNA and tRNA genes (annotation as in Figure 1C). (**F**) DRIP-qPCR as in C) targetting two tRNA and two snRNA genes. Error bars show SEM of two independent replicates.

Given the large number of predicted DRIP-seq enriched regions, which massively exceed the number of *T. brucei* loci predicted to form R-loops (Figure 1B) (80), we examined the mapping in more detail to ask about read distribution. Within the RNA Pol II PTUs, DRIP-seq enrichment was clearly non-random, with a pronounced focus on regions separating predicted coding sequences (CDS; Figure 1C); this distribution is examined in more detail below. Comparing levels of DRIP-seq enrichment relative to predicted repeated sequences (91) further suggested a non-random distribution (Supplementary Figure S2), since many regions with higher levels of enrichment coincided with clusters of repeats. Amongst such repetitive regions were the *T. brucei* centromeres, which have been mapped to date in eight of the 11 diploid megabase chromosomes (92). DRIP-seq signal was strongly enriched at the A-T rich repeats found at the centromeres (Figure 2A) in both the *Tbrh1*–/– mutants and WT cells (Figure 2A,B; Supplementary Figure S3), indicating centromeres are a focus of R-loop formation. In common with other eukaryotes, *T. brucei* rRNA genes are transcribed by RNA Pol I and organized as gene arrays. Here, although limited DRIP-seq signal peaks were observable in WT cells, pronounced signal was apparent across the rRNA locus in *Tbrh1*–/– mutants (Figure 3A–C). To ensure R-loops were being mapped, DRIP-qPCR was performed using two independently generated DRIP samples of each cell type, and in each case on-bead treatment with *E. coli* RNase HI (EcRHI) was performed in parallel with the IP reactions (Figure 3C). EcRHI treatment reduced the amount of DNA immunoprecipitated by the S9.6 antibody in both the WT and *Tbrh1–/–* DRIP samples when targeting the rRNA Pol I promoter, 5.8S or 28.S rRNA sequences (Figure 3C). In addition, in each case the amount of precipitated DNA was higher in the *Tbrh1–/–* cells than WT, in agreement with DRIP-seq mapping at this locus. Taken together, these data indicate rRNA arrays are a conserved location of R-loops (24,86). Very recently, histone H3 occupancy has mapped across the 11 megabase chromosomes of *T. brucei* (63) and comparison of histone H3 and DRIP-seq mapping indicated R-loops were readily detected *Tbrh1–/–* cells at rRNA regions where histone occupancy is lower (Figure 3B).

In contrast with other eukaryotes, *T. brucei* protein-coding genes can also be transcribed by RNA Pol I, including procyclin and VSG. DRIP-seq enrichment was notably greater in each of these loci than in the rRNA (Figure 3A). The effects of R-loop accumulation in the VSG expression sites (ES) that are actively transcribed in BSF *T. brucei* are explored in detail elsewhere (Briggs et al, BioRxiv https://doi.org/10.1101/361451), while DRIP-seq patterns at the procyclin loci and metacyclic (m) VSG expression sites, both of which display gene expression repression in this life cycle stage (93), are shown in Supplementary Figure S4. Most DRIP-seq signal detected in the mVSG expression sites was downstream of the VSG genes, which are adjacent to the telomeric repeat (Supplementary Figure S4B), and may therefore be due to TERRA RNA derived from the active BSF VSG ES (94). In *Tbrh1–/–* cells, enriched regions were also detected upstream of the mVSG coding regions and are likely due to transcription of the normally silent mVSGs in the absence of TbRH1 (Briggs et al, BioRxiv https://doi.org/10.1101/361451). Why DRIP-seq signal was observed across the procyclin loci (Supplementary Figure S4B) is less clear.

In yeast, DRIP-seq indicates R-loops form at RNA Pol III transcribed genes and their abundance increases in yeast mutants lacking both RNase H enzymes (24). Here, DRIP-seq signal was enriched at both tRNA and snRNA genes but, paradoxically, enrichment appeared to be lower in the *Tbrh1*–/– mutants (Figure 3D, E, F). A similar DRIP-seq profile was revealed for snoRNA gene arrays (Supplementary Figure S5), which are probably transcribed by RNA Pol II (95). However, DRIP-qPCR revealed pronounced detection of tRNA and snRNA sequences after IP, with substantial increases that are EcRH1-sensitive in the *Tbrh1–/–* parasites (Figure 3F). Indeed, the same dichotomy between DRIP-seq levels and DRIP-qPCR was seen for at least some genes in the RNA Pol II transcribed PTUs (see below). It is therefore likely that normalization of library size during analysis of DRIP-seq mapping underestimates the abundance of R-loops at some sites in *Tbrh1–/–* cells, and so comparison of R-loop abundance relative to WT should be treated with caution. As with the rRNA loci, snRNA and tRNA sites associated with R-loops predominantly lacked histone H3 binding (Figure 3E).

Finally, DRIP-seq revealed pronounced signal enrichment at retrotransposon hotspot (RHS) genes in *Tbrh1*–/– cells (Supplementary Figure S6), with the strongest accumulation in intergenic regions between CDS. RHS genes comprise a highly abundant, variable gene family in *T. brucei* and *T. cruzi* (96,97). Members of the RHS family express nuclear proteins and are frequent targets for transposable elements, though their functions remain elusive. Thus, though DRIP-seq enrichment here may be related to transposable elements being targets for R-loop formation in yeast (24) and plants (51), RHS localization may indicate kinetoplastid-specific R-loop functions. Here again, RHS gene arrays were markedly depleted for histone H3 (Supplementary Figure S6).

### Most *T. brucei* R-loops co-localize with sites of multigenic transcript processing

**Figure 4.**
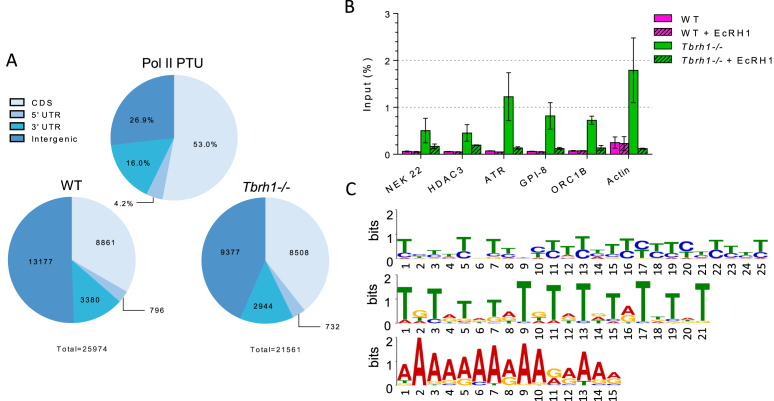
R-loops within RNA Pol II polycistronic transcription units are predominantly intergenic. (**A**) Analysis of R-loop locations in WT and *Tbrh1–/–* cells showing DRIP enriched region data sets within the Pol II PTUs (lower charts) relative to the sequence composition of Pol II PTUs (upper chart); regions are annotated according to the key. (**B**) DRIP-qPCR, as in Figure 3E, targeting the CDS of six RNA Pol II transcribed genes: NEK22 (Tb927.2.2120), HDAC3 (Tb927.2.2190), ATR (Tb927.11.14680), GPI-8 (Tb927.10.13860), ORC1B (Tb927.9.2030) and actin. (**C**) Three motifs identified by MEME analysis of WT enriched regions localized to within the Pol II PTUs.

As discussed above, DRIP-seq enrichment was most abundant throughout the RNA Pol II PTUs, with DRIP-seq signal in WT and *Tbrh1*–/– cells most pronounced in inter-CDS regions (Figure 1C), a pattern found consistently in all biological DRIP-seq replicates (Supplementary Figure S7). Quantifying the extent of signal enrichment across the all RNA Pol II transcribed genes in PTUs confirmed this (Figure 4A): in WT cells and in *Tbrh1*–/– mutants only 34% and 39%, respectively, of enriched regions localized to CDS (Figure 4A), despite these sequences comprising 53% of the PTUs. DRIP-qPCR targeting six RNA Pol II transcribed genes, including treatment with EcRHI, showed increased detection of RNA–DNA hybrids in *Tbrh1–/–* parasites compared to WT (Figure 4B), which contrasts with the limited changes in read depth in the DRIP-seq mapping (Figure 1C, Supplementary Figure S7). Hence, a potentially widespread increase in R-loops may occur across the RNA Pol II transcribed PTUs in *Tbrh1–/–* cells and was not observed with DRIP-seq due to normalization of library size. Nonetheless, such a change had no effect on growth (Briggs et al, BioRxiv https://doi.org/10.1101/361451) and a very limited effect on gene expression, since RNA-seq comparing WT and *Tbrh1*–/– cells found only five genes with clearly changed abundance, three of which were VSGs (Supplementary Figure S8A). RT-qPCR targeting RNA Pol II genes only revealed a slight change in expression of one gene, GPI-8, in *Tbrh1–/–* cells relative to WT (Supplementary Figure S9). As this gene contributes to generation of the GPI anchor that links VSG proteins to the cell membrane and *Tbrh1–/–*parasites undergo changes in VSG expression (Briggs *et al.*, BioRxiv https://doi.org/10.1101/361451), this slight increase may not be the result of increased R-loops at the coding region. Indeed, plotting of DRIP signal over genes that appeared to have intra-CDS DRIP-seq enrichment and those that do not, showed little difference in DRIP profile, indicating intra-CDS R-loops are weakly enriched compared to those at the flanking sites (Supplementary Figure S9A). Further examination of the intra-CDS R-loop positive genes did not reveal a clear pattern: base composition, CDS length, mRNA half-life and UTR length appeared indistinguishable from genes without detectable intra-CDS R-loops (Supplementary Figure S9B, C); and, for protein-coding genes, no clearly enriched GO terms could be found in the cohort (Supplementary Table S1). Thus, unlike in *A. thaliana* (51) R-loops in *T. brucei* appear to be mainly excluded from RNA Pol II-transcribed coding sequence and, when present there, cannot easily be assigned a function.

**Figure 5.**
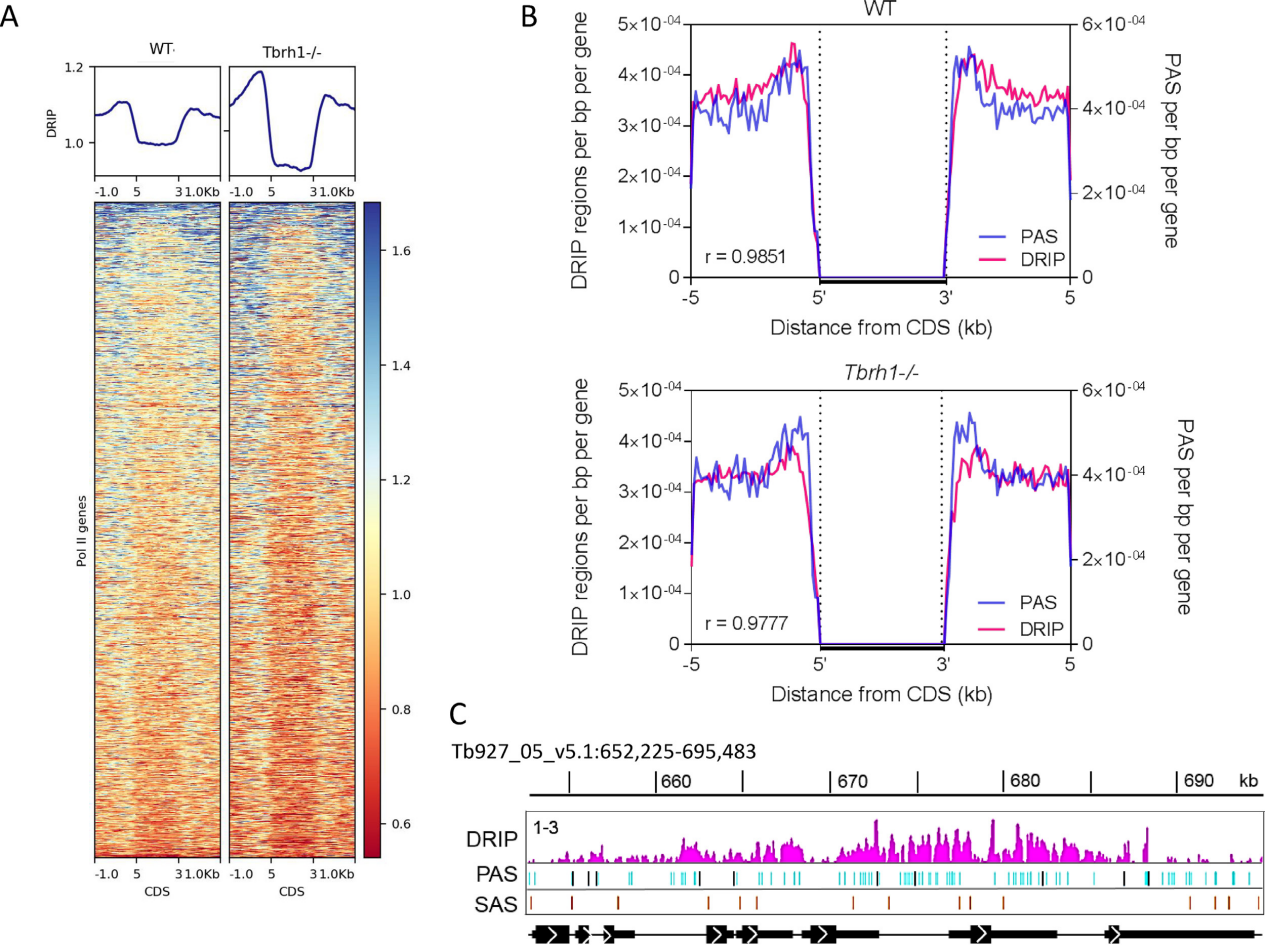
R-loop accumulation in RNA Pol II transcription units is strongly associated with polyadenylation sites. (**A**) Metaplots and heatmaps of WT and *Tbrh1–/–* DRIP signal over the CDS ±1 kb of each Pol II transcribed gene. (**B**) Metaplots of the number of UTR- or intergenic-associated DRIP enriched regions (red) and PAS (blue) regions per bp for each Pol II CDS ±5 kb for WT (upper) and *Tbrh1–/–* (lower) DRIP-seq data. (**C**) Prepresentative screenshot of WT DRIP signal (pink) relative to mapped PAS (blue; predominantly used PAS in dark blue) and SAS (orange) locations in a region of chromosome 5; CDS and DRIP-seq enrichment annotations are as shown as in Figure 1.

To examine the basis for R-loop localization within the PTUs further, motif analysis using MEME was employed, revealing enrichment of three interesting motifs in R-loop bound regions: two polypyrimidine sequences and one poly(A) tract (Figure 4C), suggesting R-loops localize to sequences associated with the *trans-*splicing and polyadenylation events needed to generate mature mRNAs from multigene RNAs. This agrees with the detailed mapping, which suggested DRIP-seq reads are most strongly enriched in intergenic sequences and untranslated regions (UTRs) in both WT and *Tbrh1*–/– cells (Figure 1C, Supplementary Figure S7). Heatmaps of DRIP-seq enrichment around every RNA Pol II gene provided confirmation, with relatively precise signal localization upstream and downstream of the CDS for probably all genes, with some evidence that enrichment increased after loss of TbRH1 (Figure 5A; DRIP-seq replicates shown in Supplementary Figure S10B), though the true extent of such a change may be confounded by data normalization during mapping. To explore this localization further we asked if the DRIP-seq enrichment pattern correlated with mapped sites of *trans*-splicing and polyadenylation (61,98), as suggested by MEME analysis. The density of polyadenylation sites (PAS) and DRIP enriched regions showed a remarkably strong correlation when analysed as density relative to distance upstream and downstream of CDS in both WT and *Tbrh1*–/– mutants (Figure 5B). Visualization of mapped reads confirmed this association, with levels of DRIP-seq enrichment notably higher in regions of clustered PAS and with patterns that follow the direction of transcription and distance from the upstream CDS (Figure 5C, Supplementary Figure S10A), suggesting R-loops form at these sites during RNA processing. Perhaps surprisingly, there was little evidence that R-loop enrichment was strongest at PAS documented as the most highly selected for a given gene (Figure 5C, Supplementary Figure S10A, C). In contrast, DRIP-seq enrichment showed a less clear association with mapped splice acceptor sites (SAS), whether predominantly used or not (Figure 5C, Supplementary Figure S10D).

**Figure 6.**
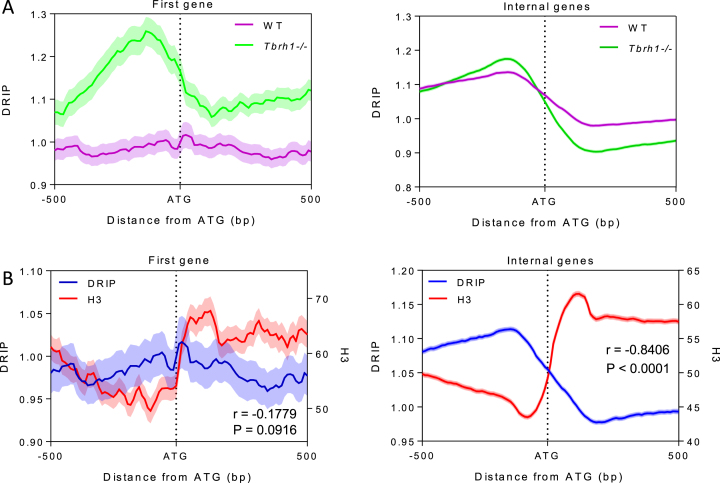
R-loop accumulation is the mirror of nucleosome accumulation and is dictated by RNaseH1 throughout RNA Pol II transcription units. (**A**) Metaplots of WT (pink) and *Tbrh1–/–* (green) DRIP signal over the ATG (±500 bp) of the first gene of each Pol II PTU (left) and over all other genes in the PTU (right). (**B**) Metaplot analysis of DRIP (blue) and histone H3 ChIP (red) signal over the ATG (±500 bp) of the first gene of each Pol II PTU (left) and all other Pol II transcibed genes (right).

To more precisely examine R-loop positioning relative to CDS, we plotted the level of DRIP-seq enrichment relative to the start codon of every RNA Pol II transcribed gene. To do this, we separated the genes into those predicted to be first within a PTU (*n*, 110), and therefore proximal to the transcription start sites, and all others (*n*, 8278), which are distal to the transcription start site and internal to the PTU. In both cases a pattern of DRIP-seq enrichment was revealed (Figure 6A) in which read abundance peaked upstream of the ATG and displayed a region of depleted reads downstream of the ATG. In WT cells this pattern was more pronounced for PTU-internal genes compared with the first genes in the PTU, whereas the same pattern was apparent in *Tbrh1*–/– mutants for both classes of gene (Figure 6A). Since mapping Histone H3 occupancy around RNA Pol II transcribed genes in *T. brucei* recently revealed nucleosome depletion upstream of every ATG (63), we mapped the H3 ChIP-seq of Wedel et al alongside our DRIP-seq data (Figure 6B). This analysis revealed a striking correspondence: for both the first genes of the PTUs and internal PTU genes the patterns of nucleosome depletion and R-loop accumulation upstream of the ATG closely mirrored each other; moreover, the region of R-loop depletion downstream of the ATG appeared to follow a small peak of increased nucleosome abundance. Taken together, these data reinforce the association of R-loops in *T. brucei* with sites of RNA processing and suggest RNA–DNA hybrids form at locations of ordered nucleosome positioning that might influence RNA Pol II movement to facilitate *trans*-splicing and polyadenylation. Examination of the AT and GC content of the sequences around the ATGs relative to R-loop enrichment (Supplementary Figure S11) suggested some increase in GC skew as sequences become more distal upstream and downstream of the ATG, a pattern that was more marked when analyzing the greater number of PTU-internal genes. Interestingly, slight GC and AT skew was found to be inversely correlated to WT DRIP signal around the ATG of all Pol II genes (Supplementary Figure S11), which contrasts with findings that R-loops are associated with positive GC skew at human CpG island promoters (47) and both AT and GC positive skew is associated with R-loops in the *Arabidopsis* genome (51).

### No evidence for R-loop localization at *T. brucei* DNA replication origins

**Figure 7.**
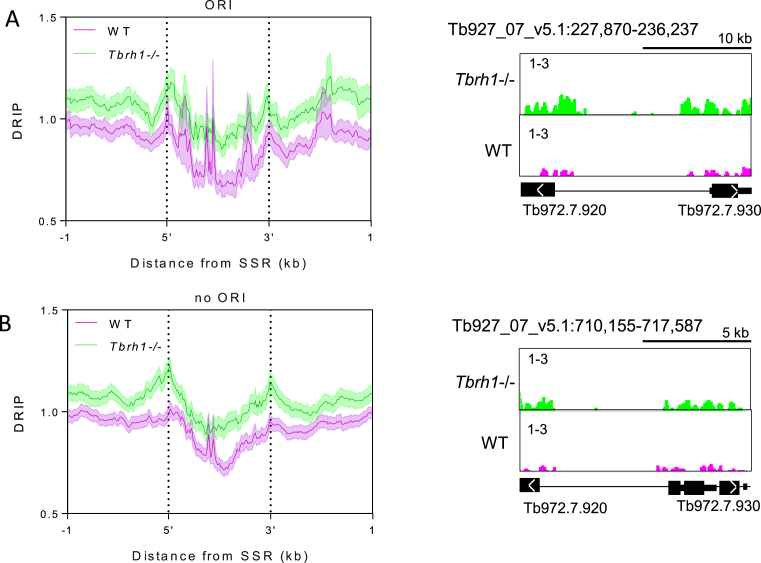
R-loop distribution is equivalent at strand switch regions at which replication initiation has been mapped, or where replication initiation has not been detected. (**A**) Metaplot analysis of WT (pink) and *Tbrh1–/–* (green) DRIP signal over replication origin (ORI)-associated SSRs (left), with a representative screenshot of one such SSR (right; gene and DRIP-seq enrichment annotations are as shown in Figure 1). (**B**) Metaplot (left) and representative screenshot (right) of SSRs where no replication origin (no ORI) activity has been detected.

Sites of DNA replication initiation, termed origins, have been mapped in a subset of SSRs in *T. brucei* by MFA-seq (67,68). What features distinguish these origin-active SSRs from origin-inactive SSRs is unclear, since one component of the *T. brucei* Origin Recognition Complex, TbORC1/CDC6, appears to map to all SSRs (69,99). In addition, no sequence features have been described that distinguish the two classes of SSRs, with the exception of highly active origins being coincident with centromeres in at least eight chromosomes (70). To ask if R-loops might represent a hitherto undetected epigenetic feature that directs origin activity, we separated SSRs into those in which replication initiation has been mapped by MFA-seq and those in which origin activity has not been detected, and examined the patterns of DRIP-seq enrichment (Figure 7). Irrespective of whether the SSRs were origin-active (Figure 7A) or –inactive (Figure 7B) a similar pattern of DRIP-seq enrichment was seen, with a striking depletion in signal around the centre of the SSR and increased signal approaching the most proximal genes. MFA-seq is unable to determine if DNA replication initiates at discrete sites within an SSR or is dispersed throughout the loci. However, as the DRIP-seq signal showed comparable levels of enrichment at the centre and CDS-proximal sites of origin-active SSRs compared with -inactive SSRs, in both WT cells and *Tbrh1–/–* mutants, it seems likely that though R-loops form within SSRs they show no differential localization that could explain the differing patterns of DNA replication initiation at these inter-PTU loci.

### R-loops are enriched at sites of transcription initiation

**Figure 8.**
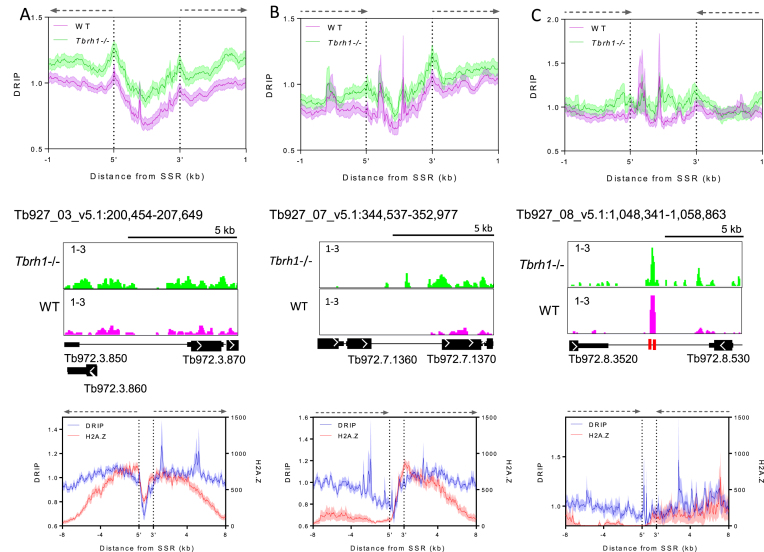
R-loop enrichment shows a greater association with multigenic transcripton initiation and associated markers than termination.The upper diagrams show metaplot profiles of WT (pink) and *Tbrh1–/–* (green) DRIP signal over divergent (**A**), head-to-tail (**B**) and convergent (**C**) SSRs (±1 kb), and the middle diagrams are representative screenshots of each (gene and DRIP-seq enrichment annotations are as shown in Figure 1, and locations of tRNA genes as red boxes). In the lower diagrams the WT DRIP signal (blue) metaplot is compared to H2A.Z ChIP signal (62) over divergent (left), head-to-tail (middle) and convergent (right) SSRs (±8 kb).

The above data suggest increased enrichment of R-loops in regions of the SSRs that are transcribed, or in elements that direct transcription initiation or termination. In other eukaryotes, R-loops have been localized to regulated promoters, suggesting roles in controlling transcription initiation (40–43), and to the ends of genes, suggesting roles in transcription termination (44–47). To ask if such R-loop roles might also act in *T. brucei*, we separated the SSRs into divergent, convergent or head-to-tail loci, where transcription and histone mapping predicts, respectively, initiation of transcription at divergent PTUs, termination of transcription at convergent PTUs, and mixed sites with transcription initiation of one PTU and termination of another. Evaluating DRIP-seq enrichment patterns in the three classes of *T. brucei* SSR suggests a role in transcription initiation but with less clear evidence for a role in termination (Figure 8). In divergent SSRs (Figure 8A) two pronounced peaks of DRIP-seq enrichment were found at the boundaries of the loci, both upstream of the first predicted genes of the two divergent PTUs. In head-tail PTUs (Figure 8B), a strong peak was again seen upstream of the first gene in the PTU, but a peak of similar magnitude was not seen around the final gene of the other PTU. Though DRIP-seq peaks could be discerned in convergent SSRs (Figure 8C), this pattern was the result of signal enrichment at tRNA genes in 2 of the 15 convergent SSRs analysed. Since the same pattern was also seen downstream of the PTUs in some head-tail SSRs (Figure 8B), and the level of DRIP-seq enrichment at the ends of the PTUs in both types of SSR appeared to be less pronounced than at the start of the PTUs (Figure 8B,C), DRIP-seq provides weaker evidence for R-loop association with sites of multigenic transcription termination compared with sites of initiation.

To further examine the association of R-loops with transcription initiation we generated metaplots of the WT DRIP-seq data at each class of SSR, extending the analysis to 8 kb upstream and downstream of the SSR boundaries, and comparing the profiles to that of previously published ChIP-seq data sets (62) of the following factors associated with transcription initiation: histone variants H2B.V and H2A.Z, histone H4 acetylated on lysine 10 (H4K10ac) and bromodomain factor BDF3 (Figure 8, Supplementary Figure S12). These plots revealed strong correlation of H2A.Z, H2B.V and H4K10ac signal with DRIP signal at the boundaries of divergent SSRs, though the correlation diminished when the plots extended into the PTUs, since here R-loops are present but enrichment of the variant and modified histones is not seen (62). The same correspondence was also seen at the transcription initiation region of head-to-tail SSRs (unlike at the termination region; see below). Enrichment of BDF3 did not correlate as well with the DRIP-seq signal, as enrichment of the bromodomain factor peaked within the SSR boundaries, and therefore upstream of the highest point of DRIP enrichment, at divergent SSRs (a pattern also seen at the 3′ boundary of head-to-tail SSRs; Supplementary Figure S12). Within convergent SSRs, as well as at the ends of PTUs in head-to-tail SSRs, there was a lack of enrichment of all four factors, reflecting the limited enrichment of DRIP-seq signal. In contrast, enrichment of the transcription termination-associated histone variants H3.V and H4.V was notably greater than that of DRIP-seq (Supplementary Figure S13), further indicating R-loops display less association with transcription termination compared with initiation in *T. brucei*.

## DISCUSSION

Work in a range of eukaryotes, including plants, insects, yeast and mammals, is revealing widespread functional roles for RNA–DNA hybrids termed R-loops. Prominent amongst these emerging activities are roles for R-loops in the control of RNA Pol II transcription initiation and termination, as well as wider localization to repeated sequences and non-coding RNA. R-loops can also cause genome instability, chromatin modifications and have been implicated in DNA replication. Much of the observations on R-loop activities are based on characterization of ‘conventional’ eukaryotic protein-coding genes, each of which is a self-contained unit bounded by a dedicated upstream RNA Pol II promoter and, downstream, by a termination region. Indeed, in some cases R-loops have been functionally associated with well understood promoters whose activity is regulated by specific RNA Pol II transcription factors. Here, we have mapped R-loop localization in the highly unconventional genome of *T. brucei*, revealing potentially diverged roles for the RNA–DNA hybrids in mRNA processing. In addition, we find pronounced R-loop localization at poorly defined and putatively unregulated RNA Pol II transcription initiation sites. Finally, we reveal conserved roles for *T. brucei* R-loops, including localization to transcribed and untranscribed repeated genes and sequences.

**Figure 9.**
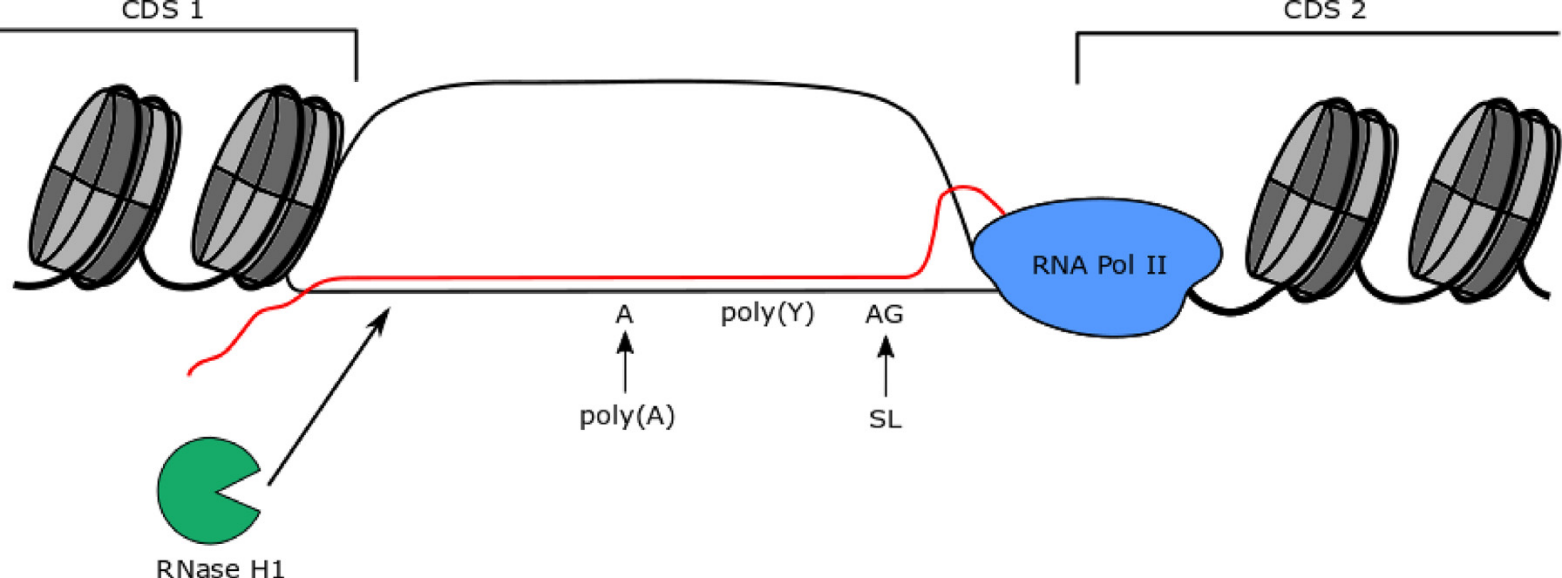
A summary of R-loop localization at mRNA processing regions in *T. brucei* multigenic RNA Pol II transcription units. Genes within Pol II PTUs are separated by UTR and intergenic DNA sequences containing sites of polyadenylation (poly(A)) and splice acceptor addtion (AG, where the splice leader (SL) 5′ cap is trans spliced), which are known to be directed by polypyrimidine (poly(Y)) tracts that are critical for correct maturation of the mRNA. R-loops containing RNA (red) putatively emerging from RNA Pol II (blue) and hybridizing to the DNA (black)form over these regions between the CDS of adjacent genes, within areas of nucleosome (grey) depletion, and are acted upon by *T. brucei* RNaseH1 (green).

Virtually every RNA Pol II transcribed gene in *T. brucei* is encoded from a multigenic transcription unit, which has resulted in the evolution of widespread and coupled trans-splicing and polyadenylation to separate adjacent genes into mature (capped and polyadenylated) mRNAs (56). The near genome-wide use of RNA Pol II multigenic gene expression is common to all kinetoplastids and its extent has no known parallel in other eukaryotic groupings. DRIP-seq mapping revealed that the most prevalent localization of R-loops in the *T. brucei* genome is within the intergenic regions between CDS in the multigenic transcription units. Though R-loops could be detected within some CDS as well, this was a minor region of DRIP-seq enrichment compared to flanking regions, which contrasts with the more equitable distribution of R-loops between gene bodies and intergenic regions observed in *A. thaliana* (51). Intra-CDS R-loops have also been described in *S. cerevisiae*, strongly correlating with highly expressed genes (52). In *T. brucei*, we have been unable to identify gene features, including mRNA half-life, mRNA length or predicted function, which dictate intra-CDS R-loop accumulation. Importantly, it is very unlikely that transcription rate can explain intra-CDS R-loops, since multigenic transcription suggests each gene within a PTU is covered by equivalent densities of RNA Pol II. Whether sequence features can alter the dynamics of RNA Pol II movement across select genes, or if some mRNAs are present for longer in the nucleus before export, allowing for increased DNA interaction, is unknown (64). Given these limitations, it is unclear if intra-CDS R-loops might function to influence gene expression. Indeed, in the absence of TbRH1, where R-loops may begin to encroach into the CDS, we see no widespread changes in *T. brucei* gene expression, though we cannot exclude the possibility that the mutant cells have adapted to loss of the RNase H. In contrast, the pronounced localization of R-loops between CDS and throughout the Pol II PTUs suggests the major genomic enrichment of R-loops in *T. brucei* is a novel association with RNA processing, reflecting the multigenic nature of kinetoplastid transcription (Figure 9).

In contrast to other eukaryotes, 5′ caps are added to kinetoplastid mRNAs by addition of a common spliced leader RNA through *trans*-splicing of multigene precursor RNA (100). Mechanistically *trans*-splicing is related to the removal of introns in other eukaryotes by *cis*-splicing, with each gene being in effect an exon. Less is understood about polyadenylation of kinetoplastid mRNAs (101,102), but the reaction follows *trans*-splicing and localization of the poly(A) tail is guided by a polypyrimidine tract and AG sequence upstream of ORFs that predominantly dictate splicing (103,104). DRIP-seq reveals abundant R-loops within the intergenic regions associated with *trans*-splicing and polyadenylation, with a strong correlation between R-loop enrichment and polyadenylation site density and proximity to the gene CDS. MEME analysis also revealed enrichment of the expected polypyrimidine tracts within Pol II PTU associated R loops, though why a poly(A) tracts was also identified is less clear, since such a feature has not been linked with *trans*-splicing or polyadenylation in PTUs. These data reveal a previously undescribed association between R-loops and mRNA processing. In yeast and humans, mapping of R-loops shows their abundance and density to be lower in intron-rich genes compared with genes lacking introns, at least in part because recruitment of the splicing machinery suppresses R-loop formation (105,106). In addition, mapping catalytically inactive RNase H1 in human cells indicates that in the rare instances where R-loops are detected within genes or at poly(A) sites, they may result from RNA processing errors and do not obviously correlate with RNA Pol II pausing sites (107). Taken together, these observations underline the novelty we see in *T. brucei*, where the predominant localization of R-loops is within RNA Pol II-transcribed multigenic units at sites where RNA processing is known to occur. Importantly, localization of R-loops to sites of polyadenylation in *T. brucei* is very unlikely to be related to the association seen in other eukaryotes between R-loops and RNA Pol II transcription termination (44–47), since at all such sites within the *T. brucei* PTUs termination must be avoided to ensure expression of downstream genes. Whether these data mean R-loops in *T. brucei*, unlike in eukaryotes with more conventional RNA Pol II genes, actively participate in the generation of mature mRNAs from a multigene precursor RNA is unclear, especially given the paucity of our understanding of polyadenylation. For instance, is it possible that RNase H cleavage of the RNA within the R-loop allows access to the polyadenylation machinery (102)? The non-essentiality of TbRH1, despite increased R-loops in the absence of the protein at RNA Pol II transcribed sites, and the lack of gene expression changes in the mutant, argues against such a crucial role. However, such an activity might be mainly assumed by RNase H2, which has not to date been functionally examined in *T. brucei*, or even shared between the two enzymes. An alternative explanation for R-loop localization within *T. brucei* PTUs might lie in the precise correlation between regions of R-loop accumulation and nucleosome depletion upstream of the ATG of all genes. This correlation may indicate RNA–DNA hybrids form at sites where RNA Pol II pauses around a well ordered nucleosome (63), in which circumstances the RNA extruded from the Pol may have greater time to bind DNA. While this does not discount the possibility that R-loops actively participate in mRNA generation, it is possible the pattern of DRIP-seq enrichment within the PTUs merely reflects the dynamics of RNA Pol II travel due to the co-ordination of mRNA processing. Indeed, ordered nucleosomes at the 5′ end of exons have been proposed to play the same role in slowing RNA Pol II and promoting cis-splicing in other eukaryotes (63,108). However, such a functional correspondence between chromatin structure and trans-splicing or *cis*-splicing cannot be readily reconciled with the strong positive correlation between *T. brucei* R-loops and mRNA processing sites compared with the poor correspondence between R-loops and cis-splicing or RNA Pol II pausing in yeast and humans (105–107). Thus, R-loop mapping revealed by DRIP-seq in *T. brucei* may reflect genuine novelty in RNA biology as a result of multigenic transcription.

SSRs separating adjacent PTUs are the sites of multigenic RNA Pol II transcription initiation and termination in *T. brucei* and all kinetoplastids. Given the precedence of R-loops contributing to both processes in other eukaryotes (8,10), DRIP-seq mapping in these loci was revealing, despite the relative lack of functional characterization of kinetoplastid RNA Pol II promoters and terminators. DRIP enrichment was most pronounced within *T. brucei* SSRs around the first gene of a PTU, indicating an association with transcription initiation. Promoters of metazoans have been separated into different classes based on transcription start site profile (109): regulated promoters have well defined start sites and mainly lack CpG islands, whereas dispersed start sites are found at promoters with CpG islands. Dispersed promoters can be ubiquitously expressed in all cell types or display developmental regulation. Though dispersed promoters are also found in *S. pombe* (110) they are less common than in metazoans, perhaps due to the lack of CpG islands. In *T. brucei*, discrete transcription start sites within SSRs appear absent (61), suggesting similarities with metazoan dispersed promoters (63), though no CpG islands or examples of transcriptional control have been detailed. R-loops have been described downstream of transcription start sites and are prevalent in human genes containing CpG islands (40,41,47), with the RNA–DNA hybrids in some at least cases contributing to the control of gene expression. R-loops have also been implicated in controlling expression from the *A. thaliana* FLC locus (43), and common features of R-loop association with metazoan transcription initiation are histone modifications and non-coding RNA. In contrast, R-loops are less clearly associated with promoters in yeast (24,28). DRIP-seq enrichment in *T. brucei* SSRs, in association with variant and modified histones, around the first PTU gene suggests R-loops might be broadly connected to transcription initiation at dispersed promoters and do not merely provide a means of transcriptional activation control. Nonetheless, the basis for R-loop deposition in *T. brucei*, and whether it is conserved with metazoans, is unclear: to date non-coding RNAs within *T. brucei* SSRs have only been described at sites where two PTUs converge (terminate) (111), the epigenetic marks localized to SSR transcription initiation sites (variant histones H2A.Z and H2B.V, histone H4K10Ac, and histone H3K4me3) (62,112) do not overlap with the chromatin signatures described at promoter-associated R-loops in humans and *A. thaliana*, and our data suggest it is not obvious that positive GC skew dictates *T. brucei* R-loop formation. In addition, the localization of R-loops within *T. brucei* SSRs, and therefore upstream of, or close to, transcription initiation, may be distinct from the predominant localization of metazoan R-loops downstream of transcription initiation. Thus, whether or not R-loops in *T. brucei* can play a role in directing transcription initiation by RNA Pol II is unclear; if R-loops play such a role, the non-essentiality of TbRH1 argues that this RNase H does not provide a critical function.

In contrast to the pronounced association of *T. brucei* R-loops with initiation of multigenic transcription, the DRIP-seq data provides less compelling evidence for a role in termination, since signal enrichment at the ends of PTUs, such as within convergent SSRs, is less pronounced. In addition, where peaks are seen at such sites they frequently localize to tRNAs. The limited enrichment we describe contrasts with the strong localization of R-loops at gene termination sites in other eukaryotes (44–47). This difference between *T. brucei* and characterized eukaryotes is perhaps surprising, since non-coding RNAs have been implicated in R-loop action during termination (44,113), and such RNAs are readily detected where multigenic transcription units converge in *T. brucei* (111). Conceivably, factors other than TbRH1, such as helicase (45,114) or cleavage complex (46) orthologues, could provide a greater role in resolving R-loops at termination sites within SSRs, meaning the DRIP-seq approach adopted here may have missed a conserved termination mechanism. Additionally, it is possible that normalization of data during the mapping falsely masked increased enrichment of DRIP-seq signal at terminators in *Tbrh1–/–* samples, as appeared to occur at RNA Pol III genes. However, a novel mechanism remains possible given the emerging role of base J (a modified base found only in kinetoplastids and close relatives) in RNA Pol II transcription termination (65,66,115,116).

Localization of R-loops to potentially all *T. brucei* SSRs at which transcription initiation occurs appears to rule out a role for the RNA–DNA hybrids in directing initiation of DNA replication, since the available mapping data suggest that only a fraction of SSRs are used constitutively as origins (67,68). In addition, origins have been described at convergent SSRs, where we see less evidence for R-loops. It is possible that R-loops contribute to recruitment of the *T. brucei* origin recognition complex, as proposed in humans (39), since ORC1/CDC6 is not limited to origin-active SSRs (67); indeed, loss of ORC1/CDC6 alters RNA levels at the ends of the PTUs, perhaps reflecting links between R-loops, replication and transcription. Nonetheless, discrete R-loop association with specific SSRs appears unable to explain why DNA replication strongly initiates at only some of the SSRs, an observation that remains unexplained in any kinetoplastid (117,118). Some caveats should be noted, however: first, it may be the case that origin-associated R-loops are specifically resolved by factors other than TbRH1, such as RNase H2; second, the approach we have taken of formaldehyde fixation may mask intra-SSR R-loops, if these regions are strongly bound by ORC. R-loops have also been implicated in unscheduled, origin-independent DNA replication at rRNA genes in yeast (38). Such a reaction would not be detected by MFA-seq mapping, which relies on isolation of S phase cells. Thus, we cannot exclude that R-loops can dictate previously undetected origin-independent DNA replication in *T. brucei*, or in other kinetoplastids. Intriguingly, though R-loops or ORC1/CDC6 binding have not been mapped to date in *Leishmania*, very widespread DNA replication initiation events, not focused on the SSRs, have been described throughout each chromosome and appear to localize at intra-CDS regions within PTUs, perhaps indicating a correlation with R-loops (119).

Repeat sequences are well-established loci for the accumulation of R-loops, and several examples of this association are seen in *T. brucei*. First, we detect DRIP-seq enrichment at the parasite's rRNA loci, as seen in both eukaryotes (86,120) and bacteria (121); as the signal is most pronounced in inter-CDS regions of WT cells, it seems likely R-loops form during transcription and not by binding of the mature RNA to DNA. Second, we detect R-loop signal at RHS-rich loci, with evidence for a dramatic increase in signal after loss of TbRH1. Here, the genesis of the R-loops is unclear; for instance, do they arise during RHS transcription, or might they be due to siRNA detected at RHS-associated retrotransposon sequences (111)? Third, we show that repeat sequences within the mapped centromeres of *T. brucei* are a very pronounced location for R-loop formation. In the three chromosomes where R-loops co-localize with centromeres containing 147 bp repeats, siRNAs may provide the RNA component of the hybrids (111). However, siRNA has not been detected at five chromosomes with centromeres that possess distinct, non-147 bp repeats (111,122), and so the genesis of R-loops at these loci is unclear. Nonetheless, R-loop association with apparently all *T. brucei* centromeres appears to reveal a conserved correlation with these DNA elements, perhaps related to chromatin modification (123) or cell cycle signalling (124), despite pronounced divergence of the kinetoplastid kinetochore complex that binds centromeres (125,126). Finally, previous work in *T. brucei* (127) and *Leishmania* (94) has revealed the existence of TERRA RNA, with over expression of TbRH1 suppressing the levels of R-loops at *T. brucei* telomere repeats. However, the extent to which (sub)telomeric R-loops might contribute to kinetoplastid biology, such as the expression or stability of telomere-proximal genes, has been little explored.

## DATA AVAILABILITY

Sequences used in the mapping have been deposited in thean European Nucleotide Archive (accession number PRJEB21868). DRIPseq analysis will be hosted at TriTrypDB (http://tritrypdb.org/tritrypdb/) in an upcoming release.

## Supplementary Material

Supplementary Data
