# Supplementary material for: Genome-wide mapping reveals conserved and diverged R-loop activities in the unusual genetic landscape of the African trypanosome genome
Source: Nucleic Acids Res. 2018 Oct 10;46(22):11789–805. doi: 10.1093/nar/gky928 (PMC6294496; doi:10.1093/nar/gky928)
Supplement: Supplementary Data [file gky928_supplemental_files.pdf]

Genome-wide mapping reveals conserved and diverged R-loop activities in the unusual genetic landscape of the African trypanosome genome

Emma Briggs, Graham Hamilton, Kathryn Crouch, Craig Lapsley and Richard McCulloch

Supplementary figure and table legends

Figure S1. *T. brucei* genome coverage by R-loops in wild type cells and in RNase H1 mutants. A) Graph showing the number of bp found to be occupied by at least one DRIP enriched region in WT and *Tbrh1-/-* cells. B) Plot showing the number of enriched regions (y-axis) of each length (x-axis) identified within WT and *Tbrh1-/-* DRIP data sets.

Figure S2. Genome-wide R-loop distribution in *T. brucei* and correlation with repetitive sequences. Screenshots of WT (pink) and *Tbrh1-/-* (green) DRIP signal over *T. brucei* megabase chromosomes 1-11; y-axis = 1-4 fold-change over input. Transcript annotations are shown above DRIP tracks, where sense-transcribed transcripts are in black and anti-sense in red. Known centromere regions are shown by peach circles over the transcript track. Lower track shows tandem repeats (navy blue).

Figure S3. DRIP-seq mapping to mapped centromeres in chromosomes 1 to 8 in *T. brucei*. Screenshots of WT (pink) and *Tbrh1-/-* (green) DRIP signal over each annotated centromere in *T. brucei* Mb chromosomes 1-8. CIR are shown in red, CDS as thick black lines and UTRs as thin black lines.

Figure S4. DRIP-seq mapping to RNA Pol I transcribed procyclin and metacyclic VSG expression sites. A) Screenshots of WT (pink) and *Tbrh1-/-* (green) DRIP-seq signal mapped to each of the five metacyclic VSG expression sites annotated in *T. brucei* strain Lister 427. Annotations as in Fig.1C; mVSG coding regions shown in red. B) Screenshot of WT (pink) and *Tbrh1-/-* (green) DRIP-seq signal mapped to a procyclin gene locus.

Figure S5. DRIP-seq mapping to snoRNA genes in *T. brucei*. Graph showing the number of DRIP enriched regions found at snoRNA loci (left) in WT and *Tbrh1*-/- cells, and a representative screenshot of the mapping (right).

Figure S6. DRIP-seq mapping to retrotransposon hotspot genes in *T. brucei*. Graph of DRIP enriched regions identified at retrotransposon hotspots (RHS) (left) in WT and *Tbrh1*-/- cells, and a representative screenshot of the mapping shown with histone H3 mapping (right).

Figure S7. R-loop distribution in RNA Pol II transcribed region s of the *T. brucei* genome. Representative screenshot of WT (pink) and *Tbrh1-/-* (green) DRIP signal enrichment at UTRs and intergenic regions within Pol II PTUs in chromosomes 2 (upper) and 7 (lower). Sequences and DRIP-seq mapping are shown as in Fig.1C, and data are shown for two biological replicates (Rep1 and Rep2).

Figure S8. Limited gene expression changes are observed after TbRH1 deletion. A) RNA-seq analysis comparing normalised gene-specific RNA reads in *Tbrh1-/-* mutants relative to WT cells. VSG genes are shown in red and all other genes as black dot ('other'); values are shown as variance-stabilising transforms of reads per kilobase of transcript per million mapped reads. B) Relative RNA abundance for a selection of RNA Pol II transcribed genes is shown by RT-qPCR in *Tbrh1-/-* parasites compared with WT (where Ct value relative to a control gene is set at 1.0); error bars show SEM from three independent replicates.

Figure S9. Analysis of R-loops that accumulate within the CDS of RNA Pol II transcribed genes.A) Metaplot of WT DRIP signal over Pol II transcribed CDS (+/- 500 bp) for those genes identified as having a WT DRIP enriched region associated with the CDS (pink, R-loop), and those without (dashed pink, No R-loop). B) Metaplot of GC (red) and AT (blue) skew over CDS (+/- 500 bp) in genes of WT cells with at least one enriched DRIP region (solid lines, R-loop), and those without (dashed lines, No R-loop). C) Box plots of CDS length, mRNA half-life, and 5' and 3' UTR lengths, of Pol II transcripts with at least one DRIP enriched region associated with the CDS in WT and *Tbrh1-/-* data sets, and those without (No R loop).

Figure S10. R-loop association with polyadenylation sites within RNA Pol II transcribed regions of *T. brucei*. A) Representative screenshot of WT (pink) and *Tbrh1-/-* (green) DRIP signal and proximity with mapped PAS (blue; predominantly used PAS in dark blue) within a Pol II PTU in chromosome 7; data are shown for two biological replicates (Rep1 and Rep2). B) Metaplots and heatmaps of WT and *Tbrh1-/-* DRIP signal over the CDS +/- 1 kb of each Pol II transcribed gene, using data from replicate 2 (Rep 1 is shown in Fig.5A). C) Metaplots and heatmaps of WT DRIP signal over the mapped dominant and non-dominant polyadenylation sites (PAS), +/- 0.5 kb, of each Pol II transcribed gene. D) Metaplots and heatmaps of WT DRIP signal over the mapped dominant and non-dominant splice acceptor sites (SAS), +/- 0.5 kb, of each Pol II transcribed gene.

Figure S11. Correlating R-loop localisation and base skew around the start codon of RNA Pol II genes in *T. brucei*. Metaplots of WT DRIP signal (pink) compared to GC (A) and AT (B) skew (blue) over the ATG (+/- 500 bp) of the first gene of each Pol II PTU (left), and over the rest of the genes with the Pol II PTU (right).

Figure S12. Correlating R-loop and transcription initiation-associated factor localisation around RNA Pol II gene SSRs in *T. brucei*. WT DRIP signal (blue) metaplot is compared to H2A.Z (A), H4K10ac (B), H2B..V (C) and BDF3 (D) ChIP-seq (red) signal over divergent (left), head-to-tail (middle) and convergent (right) SSRs (+/- 8 kb).

Figure S13. R-loop enrichment shows no correlation with histone variants associated with transcript termination. Metaplots of WT DRIP signal (blue) compared to histone H3.V (A) and H4.V (B) ChIP-seq signal (red) over divergent (left), head-to-tail (middle) and convergent (right) SSRs (+/- 8 kb).

Table S1. GO terms enriched in genes with at least one DRIP-enriched region within the CDS.

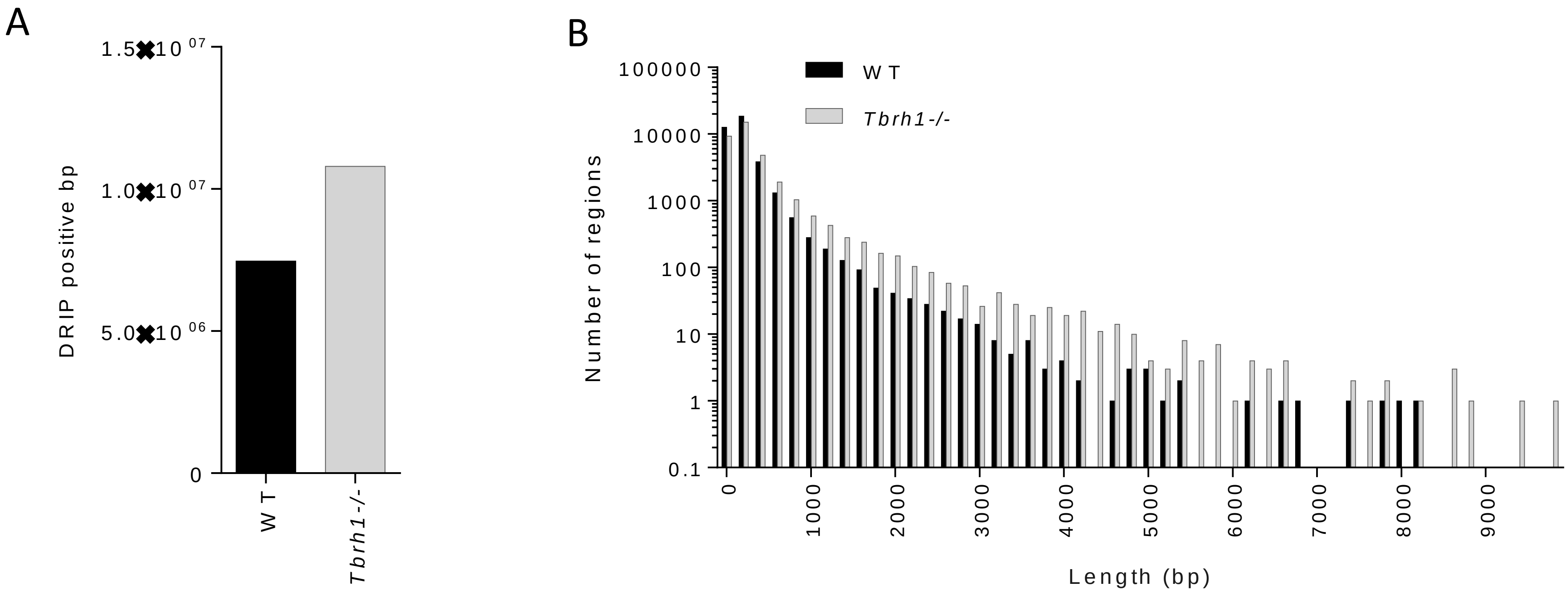
A
1.5×10^07
1.0×10^07
5.0×10^06
0
DRIP positive bp
WT
Tbrh1-/-
B
100000
10000
1000
100
10
1
0.1
Number of regions
WT
Tbrh1-/-
0
1000
2000
3000
4000
5000
6000
7000
8000
9000
Length (bp)

Fig.S1

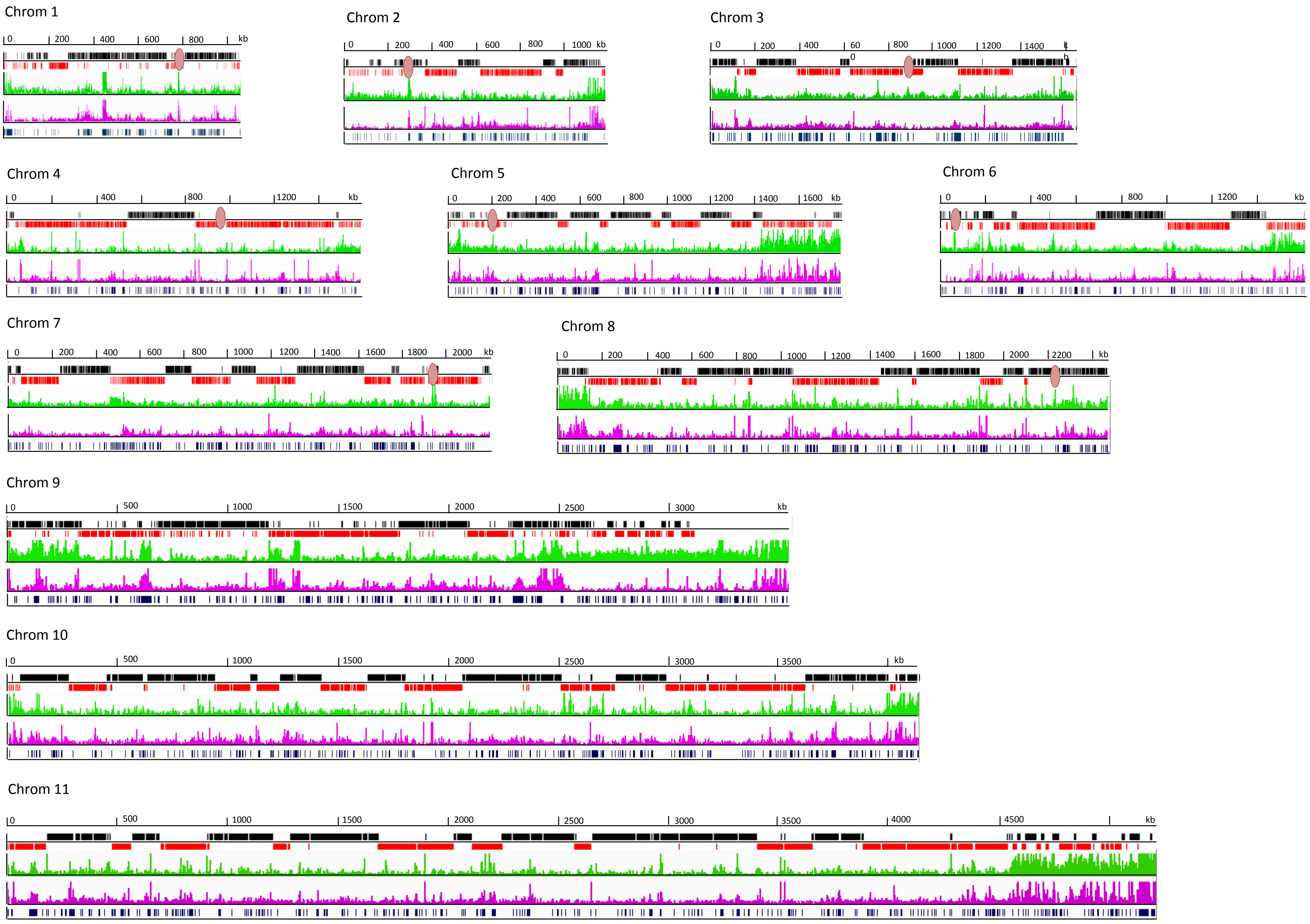
Chrom 1
0 200 400 600 800 kb
Chrom 2
0 200 400 600 800 1000 kb
Chrom 3
0 200 400 60 800 1000 1200 1400
Chrom 4
0 400 800 1200 kb
Chrom 5
0 200 400 600 800 1000 1200 1400 1600 kb
Chrom 6
0 400 800 1200 kb
Chrom 7
0 200 400 600 800 1000 1200 1400 1600 1800 2000 kb
Chrom 8
0 200 400 600 800 1000 1200 1400 1600 1800 2000 2200 kb
Chrom 9
0 500 1000 1500 2000 2500 3000 kb
Chrom 10
0 500 1000 1500 2000 2500 3000 3500 kb
Chrom 11
0 500 1000 1500 2000 2500 3000 3500 4000 4500 kb

Fig.S2

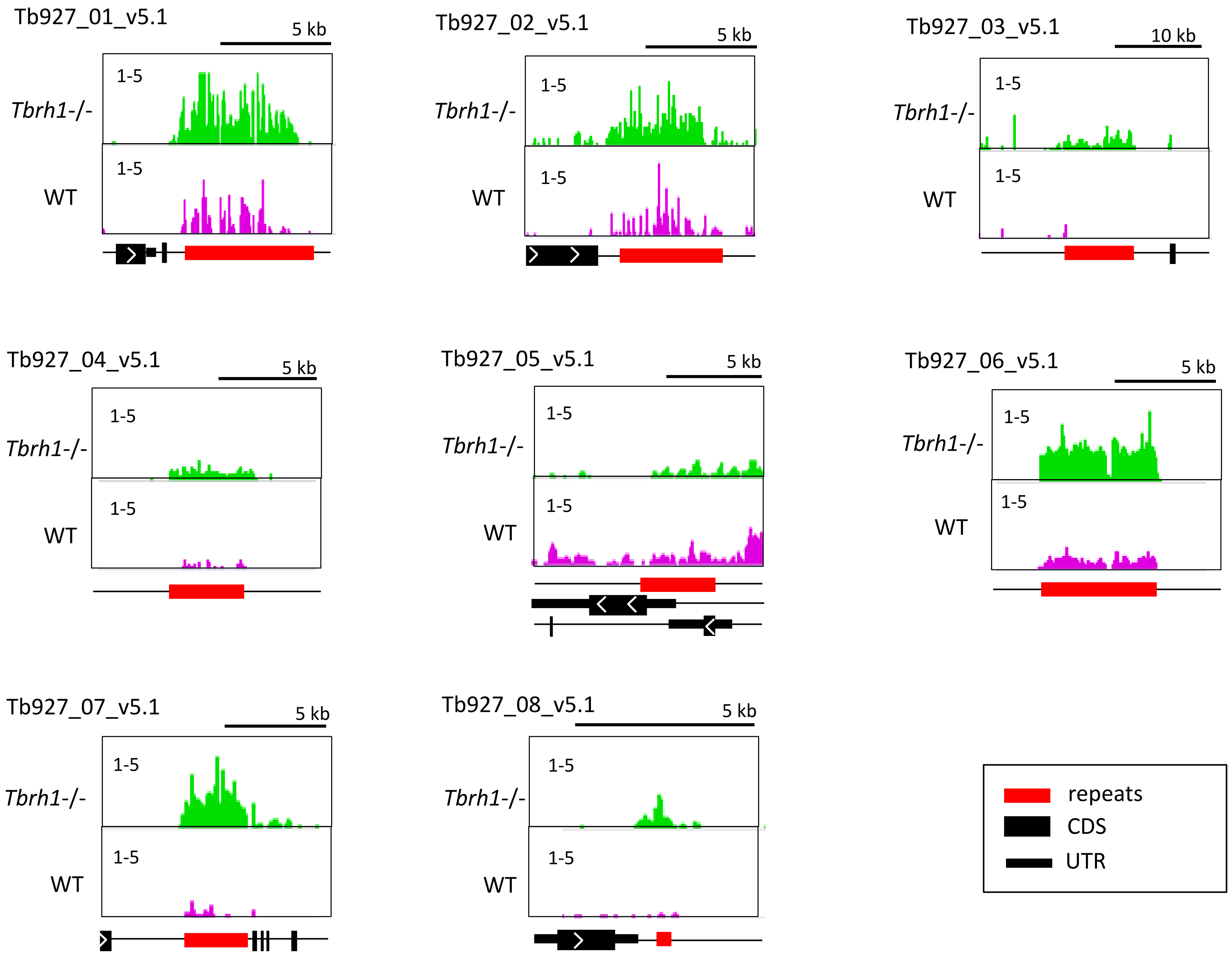
Tb927_01_v5.1
5 kb
1-5
Tbrh1-/-
1-5
WT
Tb927_02_v5.1
5 kb
1-5
Tbrh1-/-
1-5
WT
Tb927_03_v5.1
10 kb
1-5
Tbrh1-/-
1-5
WT
Tb927_04_v5.1
5 kb
1-5
Tbrh1-/-
1-5
WT
Tb927_05_v5.1
5 kb
1-5
Tbrh1-/-
1-5
WT
Tb927_06_v5.1
5 kb
1-5
Tbrh1-/-
1-5
WT
Tb927_07_v5.1
5 kb
1-5
Tbrh1-/-
1-5
WT
Tb927_08_v5.1
5 kb
1-5
Tbrh1-/-
1-5
WT
repeats
CDS
UTR

Fig.S3

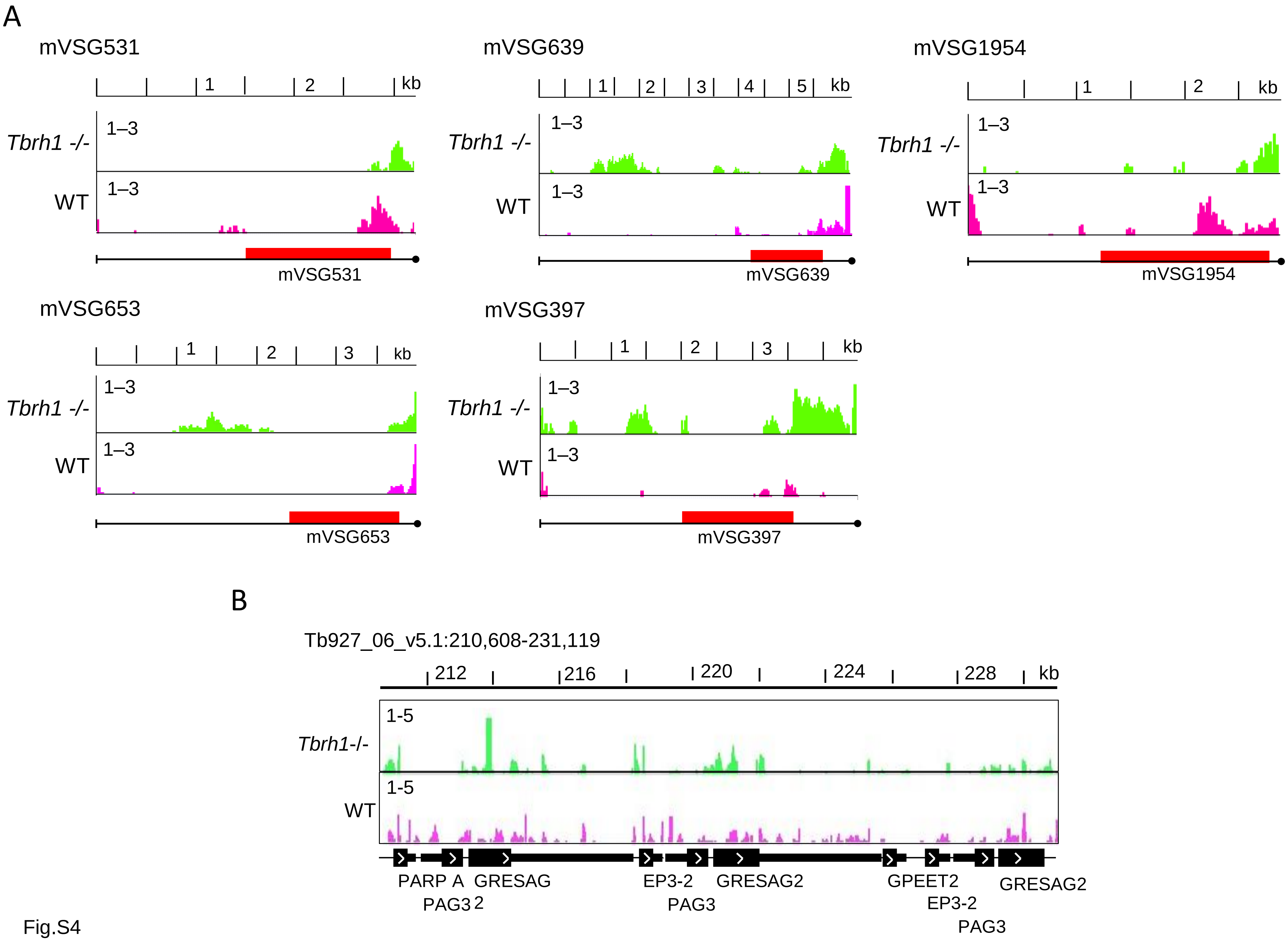
A
mVSG531
mVSG639
mVSG1954
mVSG653
mVSG397
1
2
3
4
5
kb
Tbrh1 -/-
WT
1–3
B
Tb927_06_v5.1:210,608-231,119
212
216
220
224
228
kb
Tbrh1-/-
1-5
PARP A
PAG3
GRESAG
2
EP3-2
PAG3
GRESAG2
GPEET2
EP3-2
PAG3
GRESAG2

Fig.S4

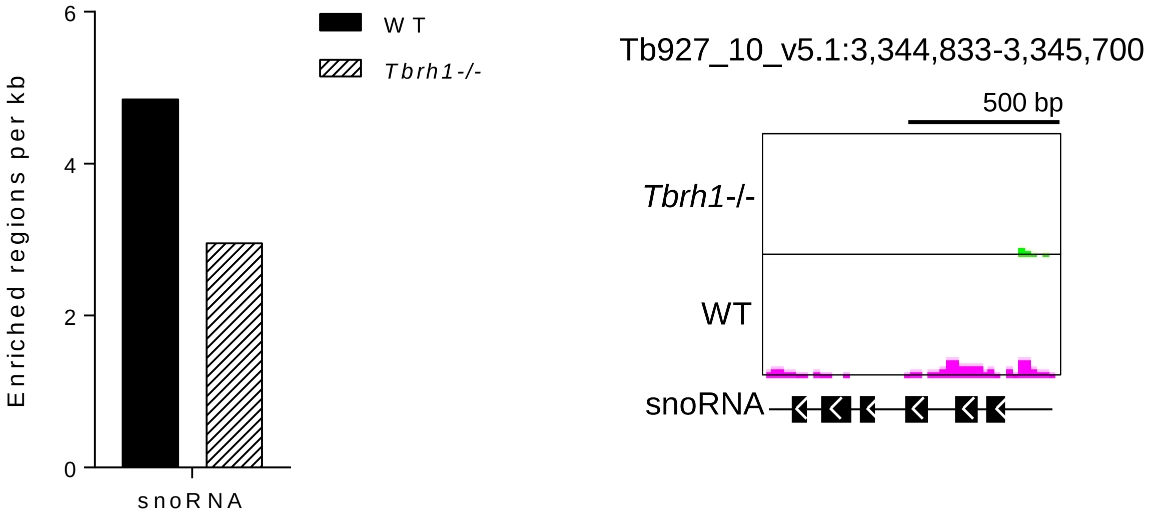

6
4
2
0
Enriched regions per kb
snoRNA
WT
Tbrh1-/-
Tb927_10_v5.1:3,344,833-3,345,700
500 bp
Tbrh1-/-
WT
snoRNA

Fig.S5

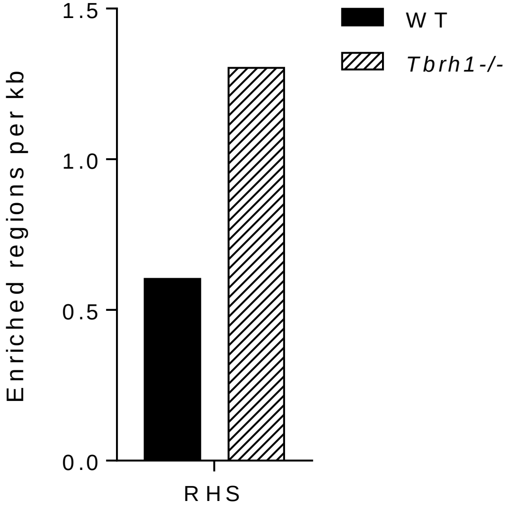

Enriched regions per kb
1.5
1.0
0.5
0.0
RHS
WT
Tbrh1-/-

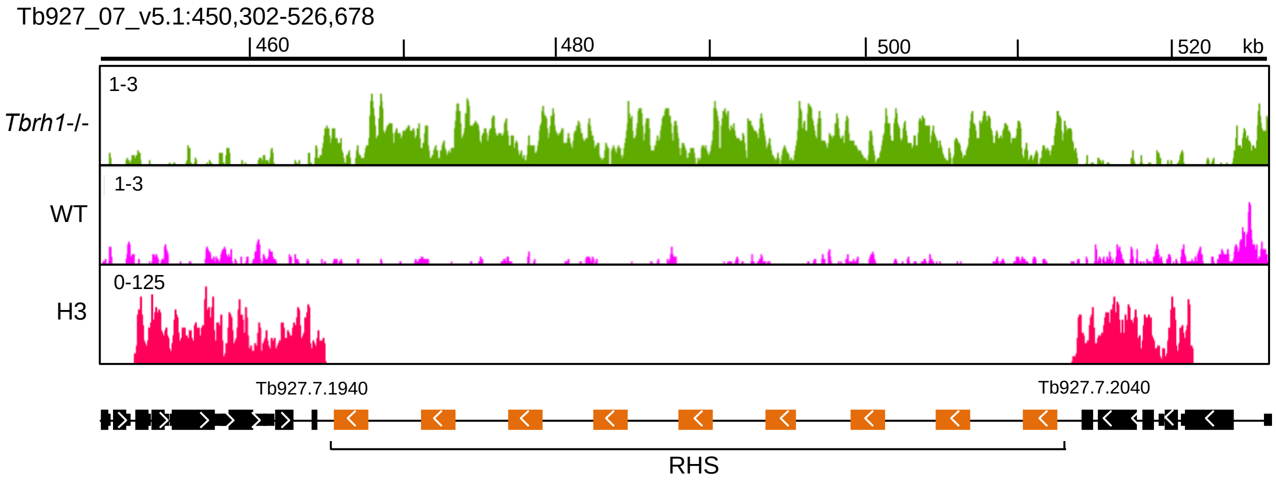

Tb927_07_v5.1:450,302-526,678
460
480
500
520
kb
Tbrh1-/-
1-3
WT
1-3
H3
0-125
Tb927.7.1940
Tb927.7.2040
RHS

Fig.S6

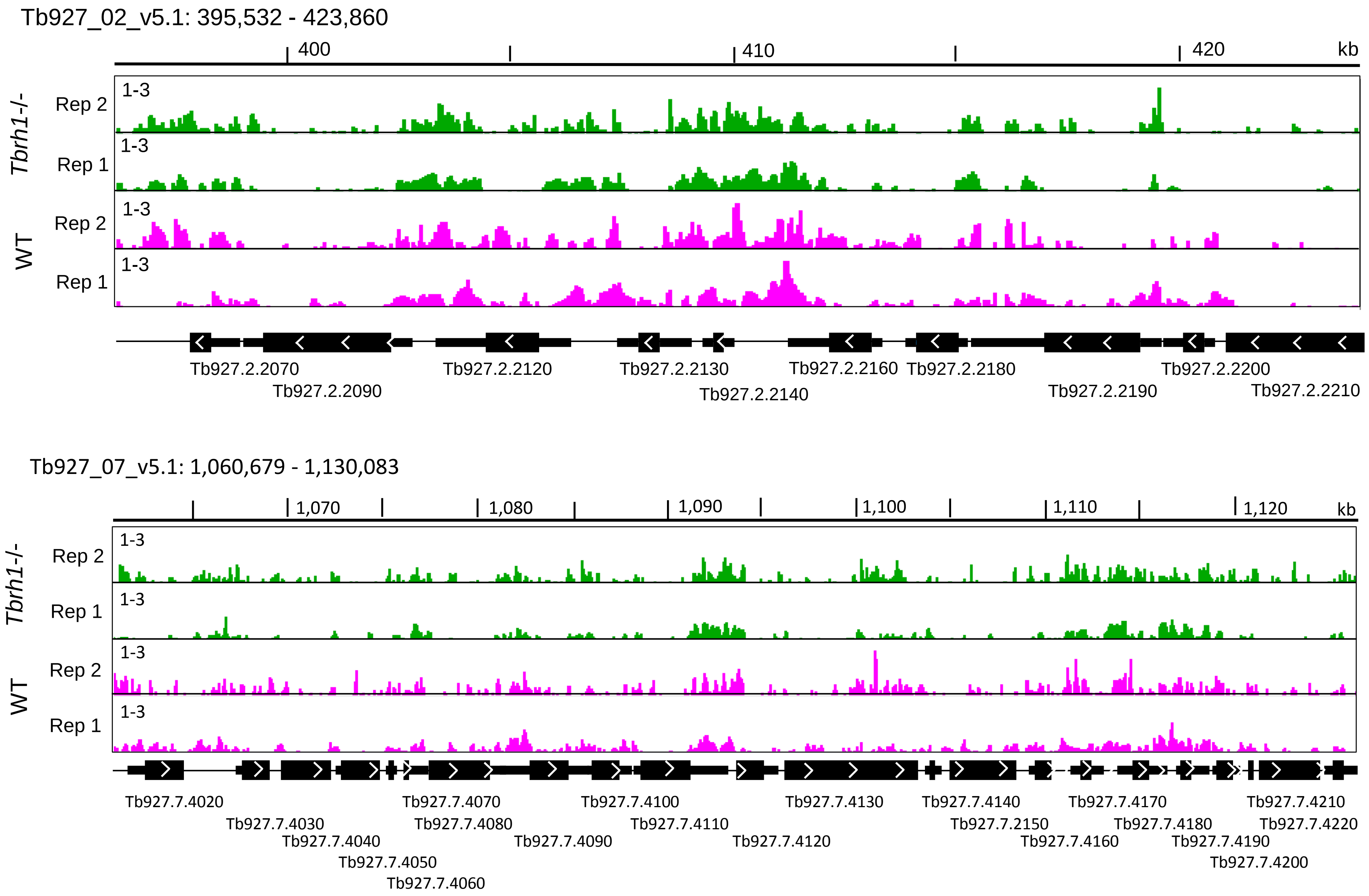
Tb927_02_v5.1: 395,532 - 423,860
400
410
420
kb
Tbrh1-/-
WT
Rep 2
Rep 1
1-3
Tb927.2.2070
Tb927.2.2090
Tb927.2.2120
Tb927.2.2130
Tb927.2.2140
Tb927.2.2160
Tb927.2.2180
Tb927.2.2190
Tb927.2.2200
Tb927.2.2210
Tb927_07_v5.1: 1,060,679 - 1,130,083
1,070
1,080
1,090
1,100
1,110
1,120
kb
Tb927.7.4020
Tb927.7.4030
Tb927.7.4040
Tb927.7.4050
Tb927.7.4060
Tb927.7.4070
Tb927.7.4080
Tb927.7.4090
Tb927.7.4100
Tb927.7.4110
Tb927.7.4120
Tb927.7.4130
Tb927.7.4140
Tb927.7.2150
Tb927.7.4160
Tb927.7.4170
Tb927.7.4180
Tb927.7.4190
Tb927.7.4200
Tb927.7.4210
Tb927.7.4220

Fig.S7

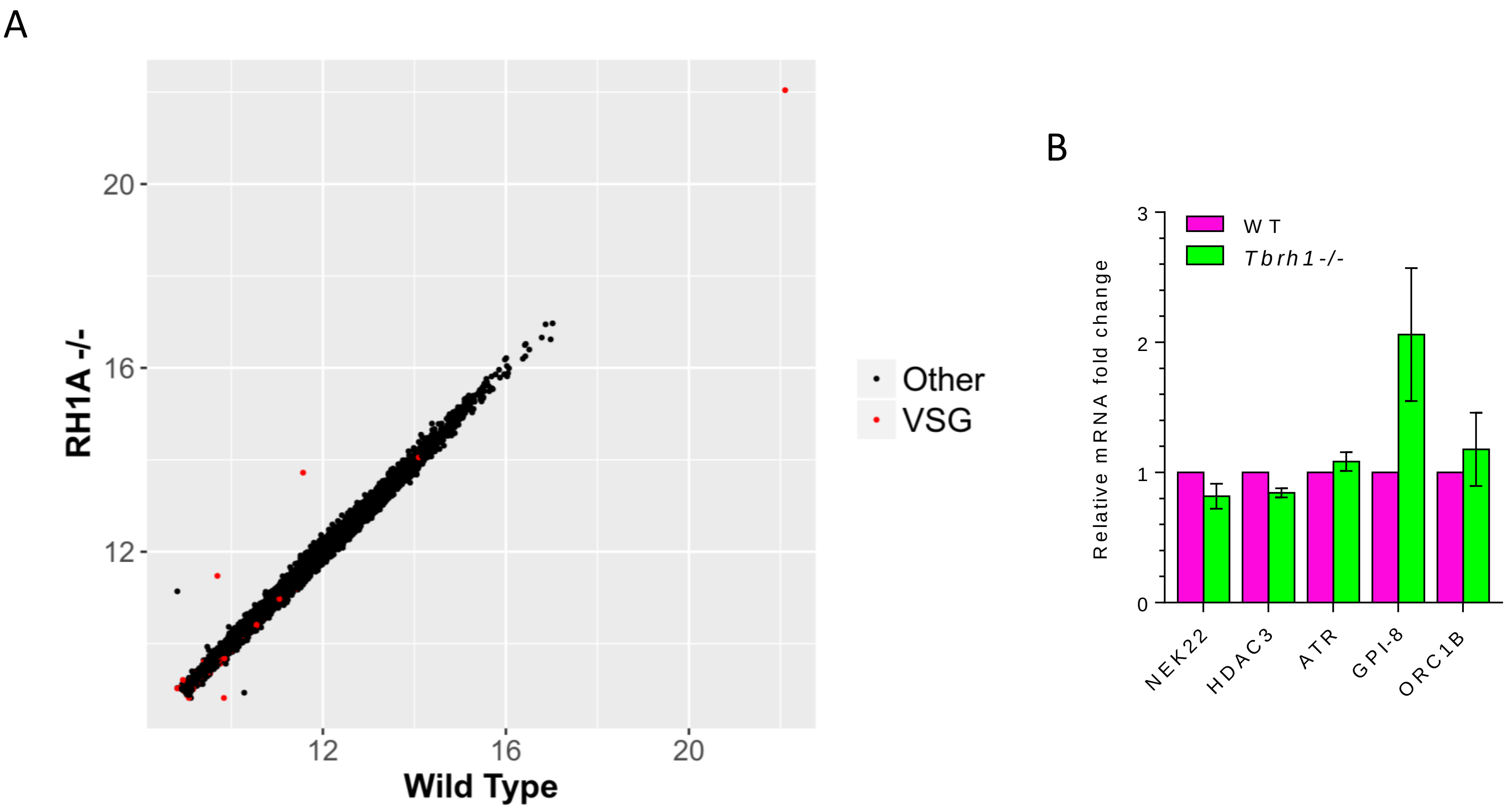
A
RH1A -/-
Wild Type
20
16
12
12
16
20
Other
VSG
B
Relative mRNA fold change
3
2
1
0
WT
Tbrh1-/-
NEK22
HDAC3
ATR
GPI-8
ORC1B

Fig.S8

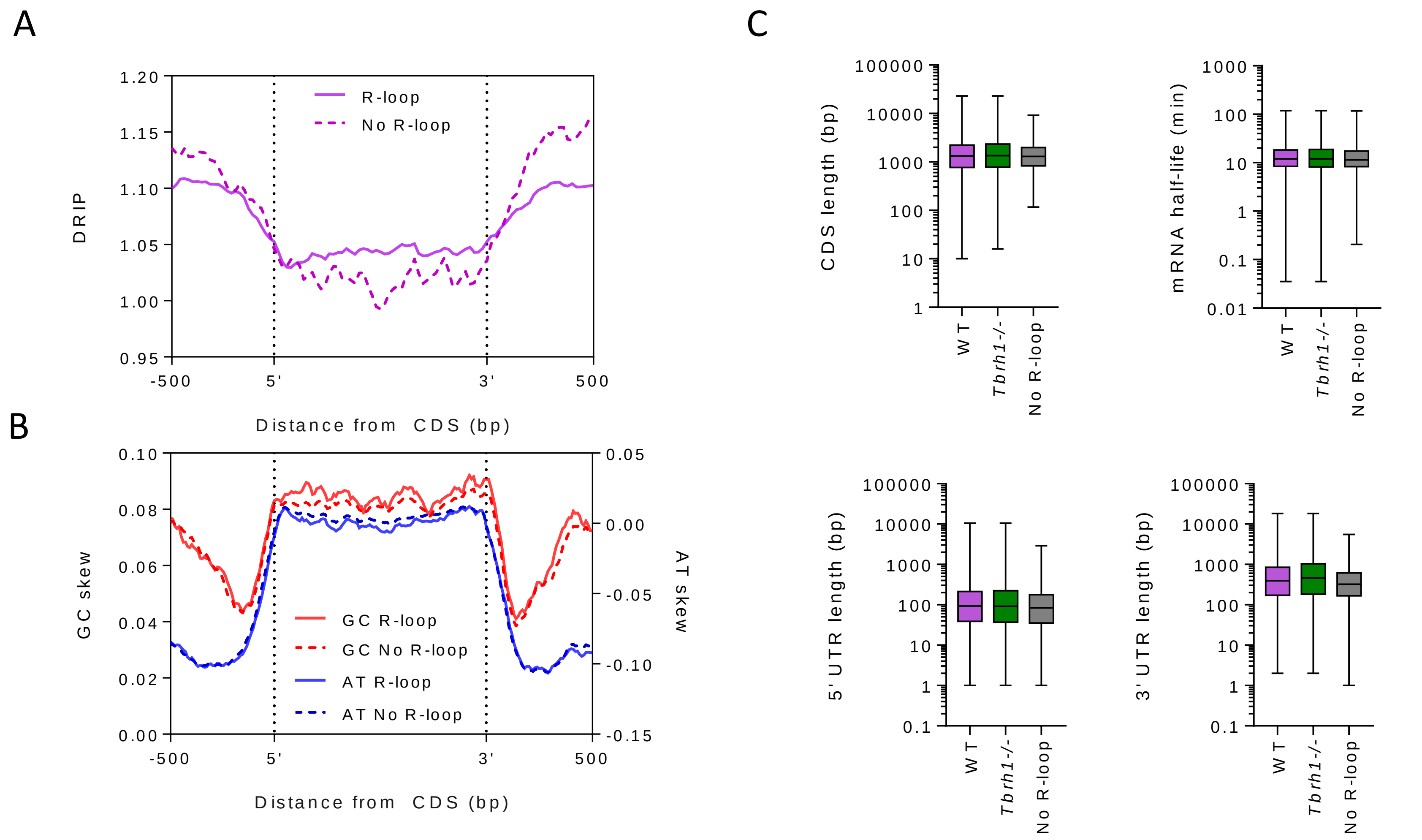
A
DRIP
R-loop
No R-loop
1.20
1.15
1.10
1.05
1.00
0.95
-500
5'
3'
500
Distance from CDS (bp)
B
GC skew
AT skew
GC R-loop
GC No R-loop
AT R-loop
AT No R-loop
0.10
0.08
0.06
0.04
0.02
0.00
0.05
0.00
-0.05
-0.10
-0.15
-500
5'
3'
500
Distance from CDS (bp)
C
CDS length (bp)
mRNA half-life (min)
5'UTR length (bp)
3'UTR length (bp)
100000
10000
1000
100
10
1
0.1
0.01
WT
Tbrh1-/-
No R-loop

Fig.S9

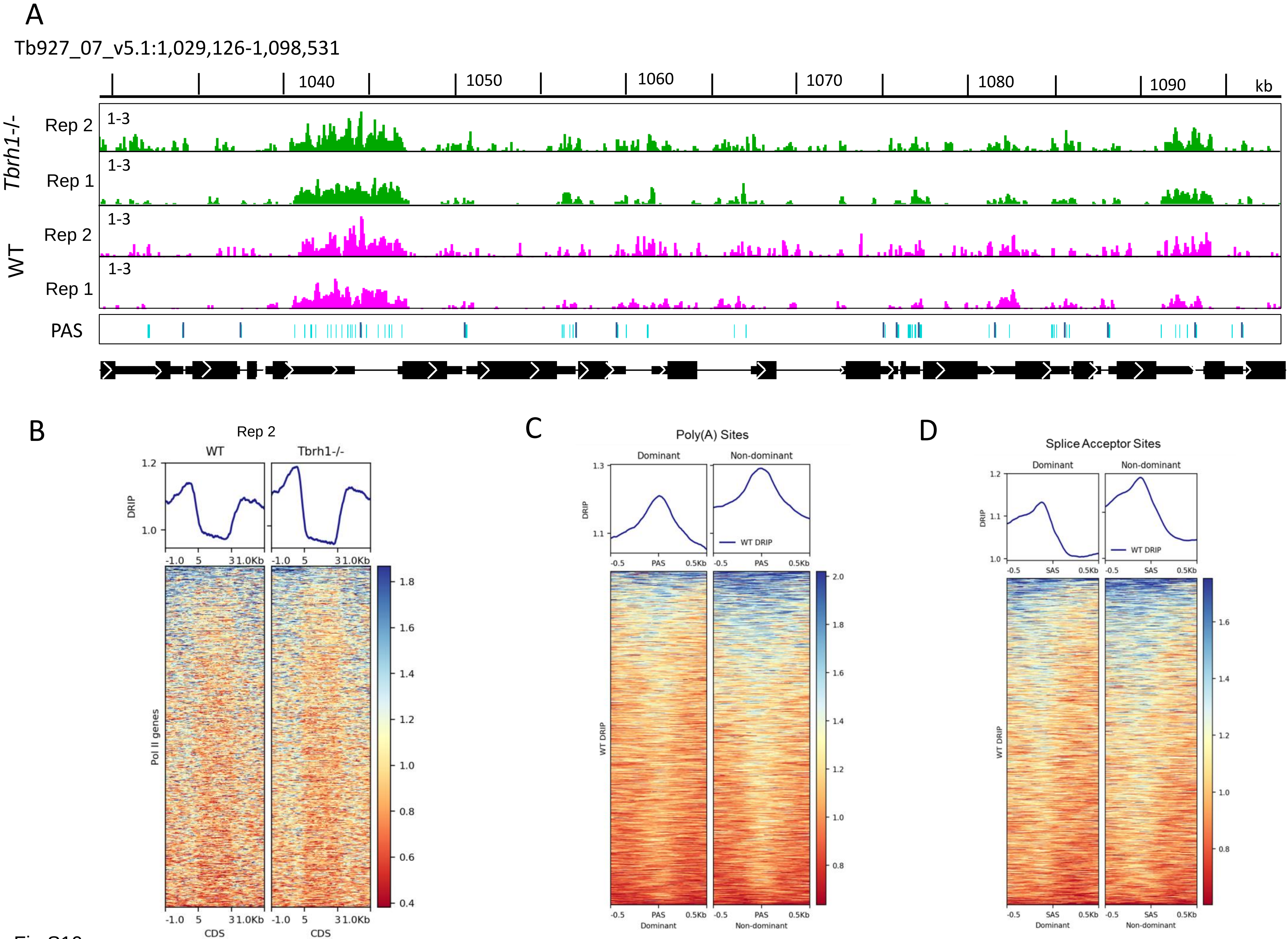
A
Tb927_07_v5.1:1,029,126-1,098,531
1040
1050
1060
1070
1080
1090
kb
Tbrh1-/-
Rep 2
Rep 1
WT
Rep 2
Rep 1
1-3
PAS
B
Rep 2
WT
Tbrh1-/-
DRIP
Pol II genes
CDS
C
Poly(A) Sites
Dominant
Non-dominant
WT DRIP
PAS
D
Splice Acceptor Sites
Dominant
Non-dominant
WT DRIP
SAS

Fig.S10

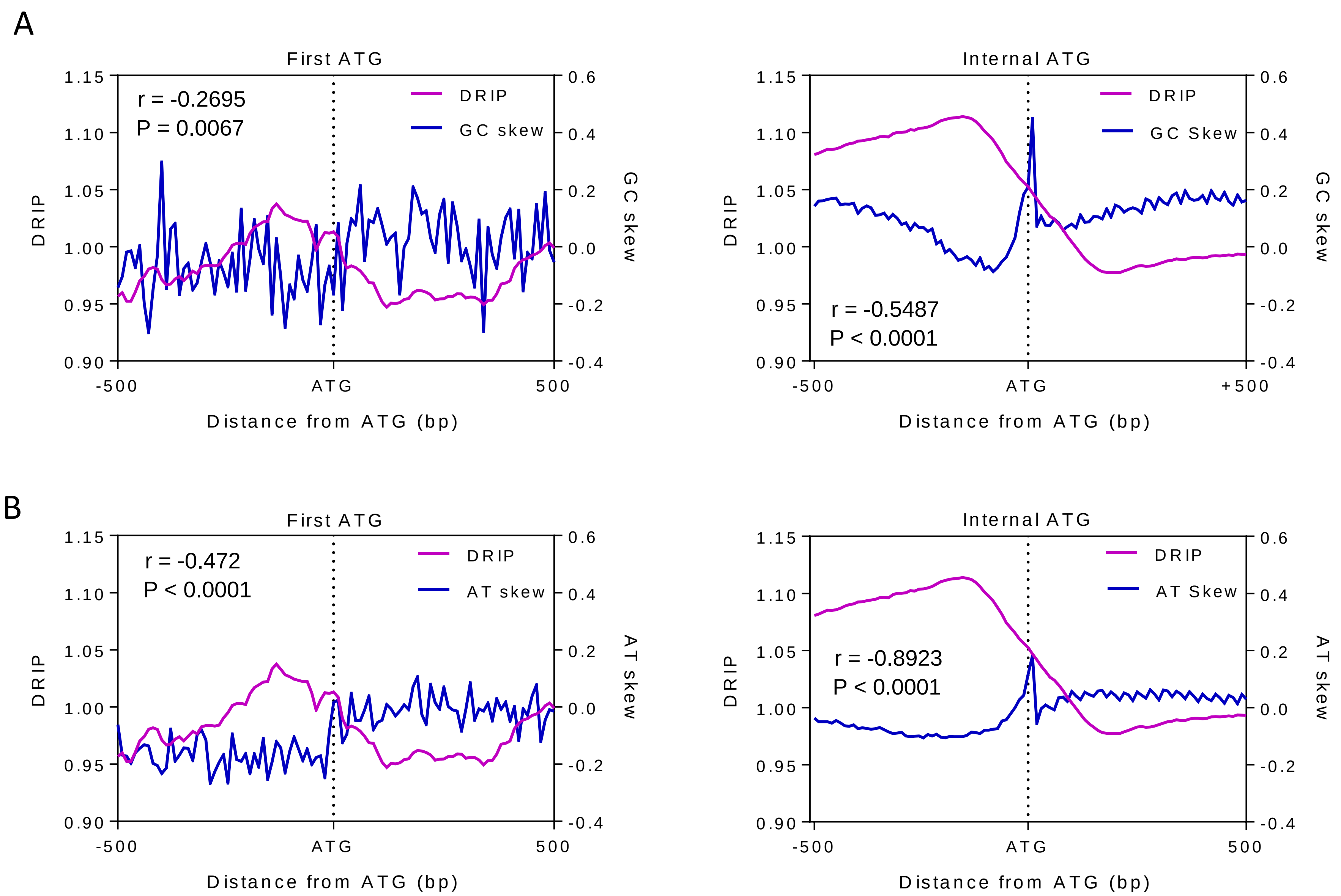
A
First ATG
r = -0.2695
P = 0.0067
DRIP
GC skew
1.15
1.10
1.05
1.00
0.95
0.90
0.6
0.4
0.2
0.0
-0.2
-0.4
-500
ATG
500
Distance from ATG (bp)
Internal ATG
DRIP
GC Skew
r = -0.5487
P < 0.0001
+500
B
First ATG
r = -0.472
P < 0.0001
AT skew
Internal ATG
AT Skew
r = -0.8923
P < 0.0001

Fig.S11

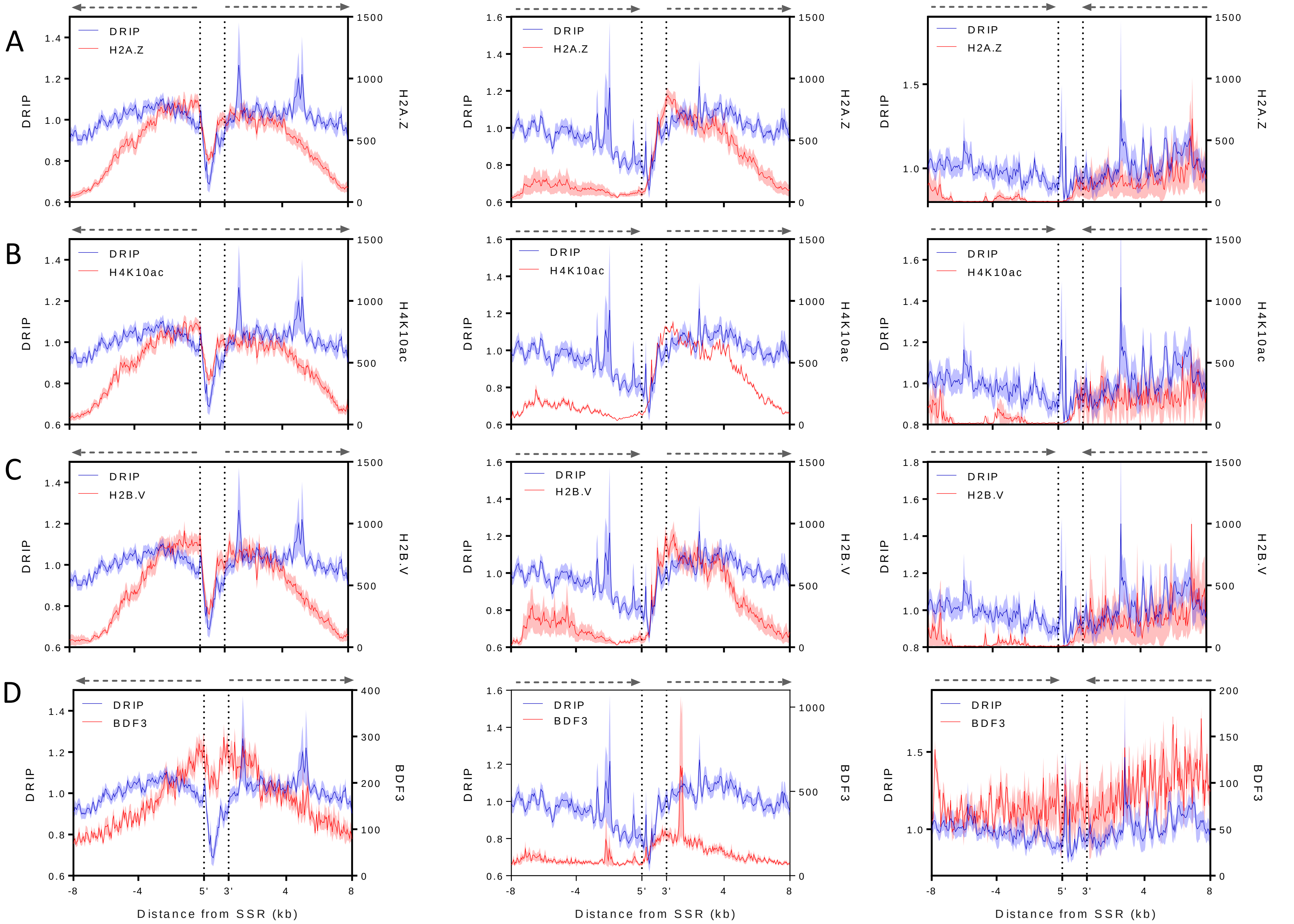

A
B
C
D
DRIP
H2A.Z
H4K10ac
H2B.V
BDF3
Distance from SSR (kb)
-8
-4
5'
3'
4
8

Fig.S12

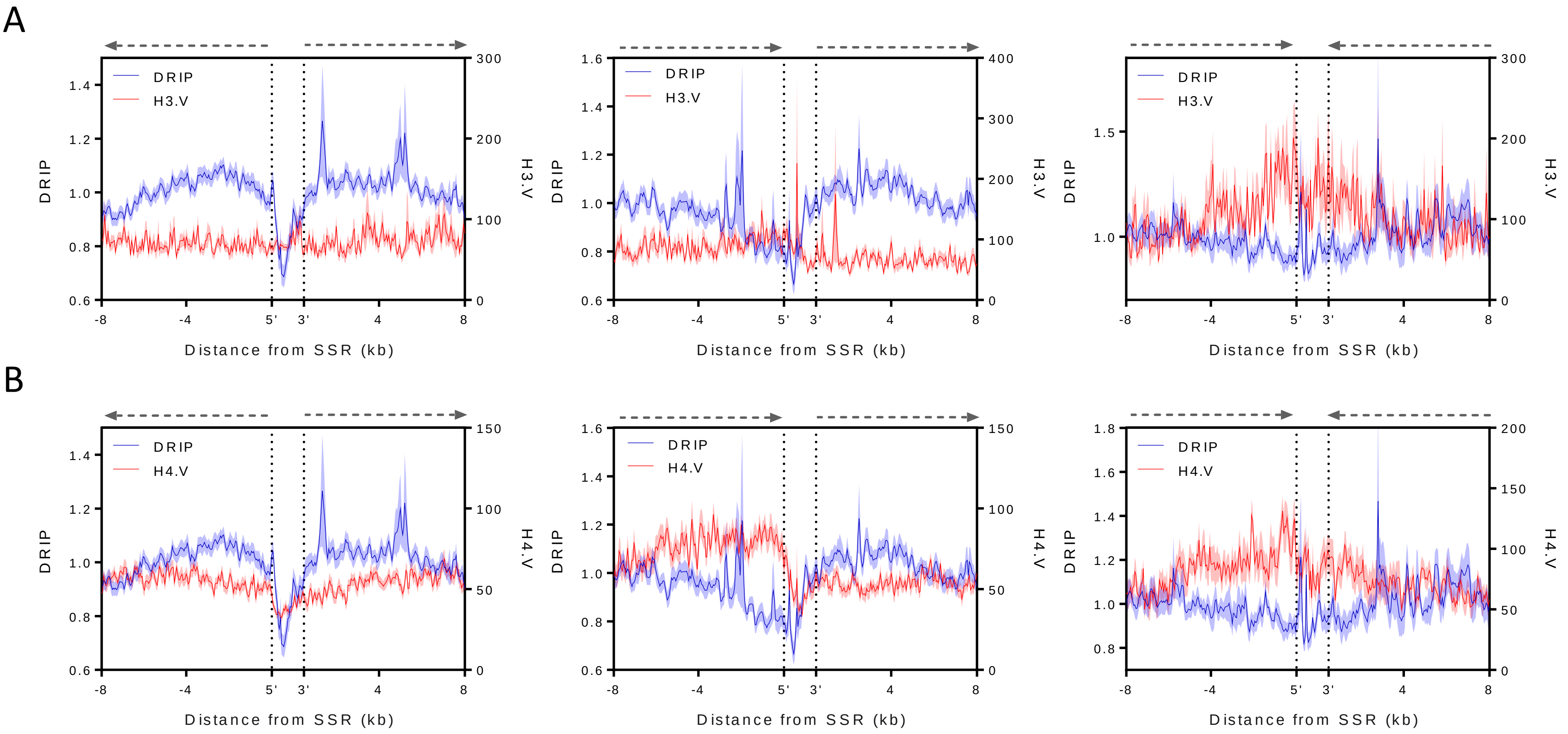

Fig.S13

ID	Name	Result count	Fold enrichment	P-value	Benjamini
GO:0005488	binding	1509	1.13	7.88E-08	3.94E-06
GO:0032559	adenyl ribonucleotide binding	397	1.32	3.16E-06	4.84E-05
GO:0030554	adenyl nucleotide binding	401	1.32	3.28E-06	4.84E-05
GO:0005524	ATP binding	395	1.32	3.87E-06	4.84E-05
GO:0032555	purine ribonucleotide binding	453	1.27	1.42E-05	6.55E-05
GO:0032549	ribonucleoside binding	452	1.26	1.55E-05	6.55E-05
GO:0001882	nucleoside binding	452	1.26	1.55E-05	6.55E-05
GO:0032550	purine ribonucleoside binding	452	1.26	1.55E-05	6.55E-05
GO:0001883	purine nucleoside binding	452	1.26	1.55E-05	6.55E-05
GO:0017076	purine nucleotide binding	457	1.26	1.59E-05	6.55E-05
GO:0097367	carbohydrate derivative binding	459	1.26	1.60E-05	6.55E-05
GO:0032553	ribonucleotide binding	459	1.26	1.60E-05	6.55E-05
GO:0035639	purine ribonucleoside triphosphate binding	451	1.26	1.70E-05	6.55E-05
GO:0003674	molecular function	2075	1.07	3.08E-05	0.000109916
GO:0000166	nucleotide binding	490	1.23	6.03E-05	0.00018835
GO:1901265	nucleoside phosphate binding	490	1.23	6.03E-05	0.00018835
GO:0036094	small molecule binding	494	1.23	6.59E-05	0.000193732
GO:0043168	anion binding	480	1.23	7.04E-05	0.000195516
GO:0005515	protein binding	636	1.18	0.000103838	0.000273259
GO:0097159	organic cyclic compound binding	842	1.14	0.000176392	0.000419981
GO:1901363	heterocyclic compound binding	842	1.14	0.000176392	0.000419981
GO:0043167	ion binding	678	1.16	0.000262957	0.000597629
GO:0016887	ATPase activity	112	1.44	0.001624295	0.003531077
GO:0042623	ATPase activity, coupled	87	1.49	0.002549561	0.005311585
GO:0016462	pyrophosphatase activity	200	1.26	0.004109398	0.007902688
GO:0016818	hydrolase activity, acting on acid anhydrides, in phosphorus-containing anhydrides	200	1.26	0.004109398	0.007902688
GO:0017111	nucleoside-triphosphatase activity	195	1.27	0.004382276	0.008115326
GO:0016817	hydrolase activity, acting on acid anhydrides	201	1.26	0.004966523	0.008868791
GO:0008026	ATP-dependent helicase activity	43	1.66	0.008623755	0.014372925
GO:0070035	purine NTP-dependent helicase activity	43	1.66	0.008623755	0.014372925
GO:0016787	hydrolase activity	421	1.14	0.009754508	0.015733077
GO:0016773	phosphotransferase activity, alcohol group as acceptor	151	1.25	0.015078862	0.023560721
GO:0004386	helicase activity	51	1.49	0.018276414	0.027235004
GO:0004672	protein kinase activity	122	1.27	0.018519803	0.027235004
GO:0016301	kinase activity	161	1.23	0.020322907	0.028621349
GO:0004016	adenylate cyclase activity	41	1.56	0.020607371	0.028621349
GO:0003729	mRNA binding	110	1.28	0.022840644	0.030393689
GO:0003774	motor activity	59	1.42	0.023099204	0.030393689
GO:0004674	protein serine/threonine kinase activity	75	1.35	0.024219967	0.030838196
GO:0009975	cyclase activity	41	1.53	0.024670556	0.030838196
GO:0003723	RNA binding	249	1.16	0.027699753	0.032811201
GO:0044822	poly(A) RNA binding	110	1.27	0.027812196	0.032811201
GO:0022804	active transmembrane transporter activity	69	1.36	0.028217632	0.032811201
GO:0008094	DNA-dependent ATPase activity	21	1.82	0.031811929	0.035760812
GO:0003777	microtubule motor activity	55	1.4	0.032184731	0.035760812
GO:0003676	nucleic acid binding	436	1.11	0.033776143	0.036562734
GO:0016772	transferase activity, transferring phosphorus-containing groups	228	1.16	0.03436897	0.036562734
GO:0005215	transporter activity	138	1.21	0.036986137	0.038527226
GO:0008017	microtubule binding	44	1.42	0.044181551	0.045083215
GO:0003824	catalytic activity	1000	1.06	0.048957109	0.048957109

Table S1.
